# PARK7 deficiency inhibits fatty acid β‐oxidation via PTEN to delay liver regeneration after hepatectomy

**DOI:** 10.1002/ctm2.1061

**Published:** 2022-09-23

**Authors:** Xiaoye Qu, Yankai Wen, Junzhe Jiao, Jie Zhao, Xuehua Sun, Fang Wang, Yueqiu Gao, Weifeng Tan, Qiang Xia, Hailong Wu, Xiaoni Kong

**Affiliations:** ^1^ Department of Liver Surgery, Renji Hospital, School of Medicine Shanghai Jiao Tong University Shanghai China; ^2^ Central Laboratory Department of Liver Diseases ShuGuang Hospital Affiliated to Shanghai University of Chinese Traditional Medicine Shanghai China; ^3^ Shanghai Key Laboratory of Molecular Imaging Shanghai University of Medicine and Health Sciences Shanghai China

**Keywords:** hepatocyte‐specific knockout, liver regeneration, *Park7/Dj1*, transient regeneration–associated steatosis

## Abstract

**Background & aims:**

Transient regeneration–associated steatosis (TRAS) is a process of temporary hepatic lipid accumulation and is essential for liver regeneration by providing energy generated from fatty acid β‐oxidation, but the regulatory mechanism underlying TRAS remains unknown. Parkinsonism‐associated deglycase (*Park7*)/*Dj1* is an important regulator involved in various liver diseases. In nonalcoholic fatty liver diseased mice, induced by a high‐fat diet, *Park7* deficiency improves hepatic steatosis, but its role in liver regeneration remains unknown

**Methods:**

*Park7* knockout (*Park7* ^−/−^), hepatocyte‐specific *Park7* knockout (*Park7* ^△hep^) and hepatocyte‐specific *Park7‐Pten* double knockout mice were subjected to 2/3 partial hepatectomy (PHx)

**Results:**

Increased PARK7 expression was observed in the regenerating liver of mice at 36 and 48 h after PHx. *Park7* ^−/−^ and *Park7* ^△hep^ mice showed delayed liver regeneration and enhanced TRAS after PHx. PPARa, a key regulator of β‐oxidation, and carnitine palmitoyltransferase 1a (CPT1a), a rate‐limiting enzyme of β‐oxidation, had substantially decreased expression in the regenerating liver of *Park7* ^△hep^ mice. Increased phosphatase and tensin homolog (PTEN) expression was observed in the liver of *Park7* ^△hep^ mice, which might contribute to delayed liver regeneration in these mice because genomic depletion or pharmacological inhibition of PTEN restored the delayed liver regeneration by reversing the downregulation of PPARa and CPT1a and in turn accelerating the utilization of TRAS in the regenerating liver of *Park7* ^△hep^ mice

**Conclusion:**

*Park7*/*Dj1* is a novel regulator of PTEN‐dependent fatty acid β‐oxidation, and increasing *Park7* expression might be a promising strategy to promote liver regeneration.

## Graphical abstract

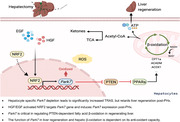

## 1 INTRODUCTION

The liver has substantial capacity to regenerate and recover from injury, which is a complex process orchestrated by multiple cytokines, growth factors and metabolic alterations.^1^ As the liver acts as the principal glucose reservoir in the body, liver mass loss after partial hepatectomy (PHx) inevitably results in hypoglycaemia, which in turn triggers a series of metabolic alterations in the liver.^2^ Among them, temporary hepatic lipid accumulation, also termed transient regeneration–associated steatosis (TRAS), is one of the most remarkable physiological alterations during PHx‐induced liver regeneration.^3^ During TRAS, the lipids accumulated in the liver are derived from the systemic lipolysis of peripheral fat stores and are rapidly utilized to produce ATP by β‐oxidation to assist in rapid liver regeneration.^3^, ^4^, ^5^ The TRAS level generally peaks at 16–24 h and declines to a low level at 48–72 h after PHx in mice.^6^, ^7^ Inappropriate lipid accumulation has been shown to impair PHx‐induced liver regeneration.^8^ Nevertheless, the regulatory factors associated with TRAS, β‐oxidation and liver regeneration remain largely unknown.

*Dj1*, also called Parkinsonism‐associated deglycase (*Park7*), was initially identified as a novel oncogene to facilitate mouse NIH 3T3 cell transformation along with H‐Ras.^9^ After decades of investigation, *Park7* has been demonstrated to possess versatile functions, including regulating androgen receptor‐dependent transcription,^10^, ^11^ antioxidant responses^12^ and intracellular signalling pathways^13^, ^14^, ^15^; scavenging reactive oxygen species (ROS)^16^; and chaperoning selected proteins.^17^ We and other groups have showed that *Park7* is associated with the pathogenesis of many liver diseases, mainly dependent on its redox function. A reactive cysteine residue at position 106 (Cys106) of PARK7, the key residue for its biological function, is oxidized under oxidative stress.^18^ *Park7* has been shown to facilitate liver progenitor cell expansion in a 3,5‐diethoxycarbonyl‐1,4‐dihydrocollidine diet‐induced liver injury murine model^19^ and promote the development of diethylnitrosamine‐induced hepatocellular carcinoma^20^; *Park7* deficiency could improve carbon tetrachloride‐induced liver fibrosis and liver ischemia–reperfusion injury.^18^, ^21^ Several previous studies have shown that *Park7* could activate several signalling pathways, such as ERK1/2 and PI3K/Akt, which are well documented to play a pro‐proliferation role in liver regeneration^22^, ^23^; however, the role of *Park7* in liver regeneration has not been investigated.

Phosphatase and tensin homolog (PTEN) is a well‐known tumour suppressor, which prevents tumour progression by negatively regulating the ERK1/2 and PI3K/Akt signalling pathways.^24^, ^25^ A recent study has demonstrated that PTEN deficiency led to enhanced hypertrophic liver growth after PHx via the promotion of β‐oxidation of fatty acids derived from TRAS.^6^ As an oncogene, *Park7* has been proved to function as a negative regulator of PTEN in many cancer types^13^, ^26^, ^27^ by either antagonizing PTEN function or inhibiting PTEN expression.^28^, ^29^ However, whether *Park7* is involved in the TRAS–β‐oxidation–liver regeneration regulatory axis remains unknown.

In the present study, we showed that hepatectomy‐activated nuclear factor erythroid 2–related factor 2 (NRF2) and its target gene, *Park7*. Hepatocyte‐specific *Park7* deficiency significantly increased TRAS but delayed liver regeneration after PHx. This phenomenon was related to the decreased expression of PPARa, a key transcription factor of β‐oxidation, and carnitine palmitoyltransferase 1a (CPT1a), a rate‐limiting enzyme of β‐oxidation. Mechanistically, *Park7* deficiency induced PTEN activation, which contributed to decreased fatty acid β‐oxidation during liver regeneration. Oxidized PARK7 (oxPARK7) contains Cys106 and played a major role in this process. Thus, *Park7*/*Dj1* might serve as a potential therapeutic target for promoting liver regeneration.

## 2 RESULTS

### 2.1 *Park7* is associated with liver regeneration after PHx

To investigate the possible involvement of *Park7* in liver regeneration, we first examined the hepatic PARK7 levels in wild‐type (WT) mice after PHx. Increased hepatic PARK7 expression was detected in mice 24–48 h after PHx using immunoblot analysis (Figure S1A,B). Next, we assessed human serum PARK7 levels in normal donors, who donated parts of healthy livers to their children receiving paediatric living donor liver transplants, at 24 and 72 h after left lateral lobectomy. Interestingly, the serum PARK7 levels significantly increased at both 24 and 72 h after PHx in healthy donors (Figure S1C). These results indicate that *Park7* might act as a critical target in liver regeneration after PHx.

### 2.2 Delayed liver regeneration in *Park7* knockout (*Park7* ^−/−^) mice

**FIGURE 1:**
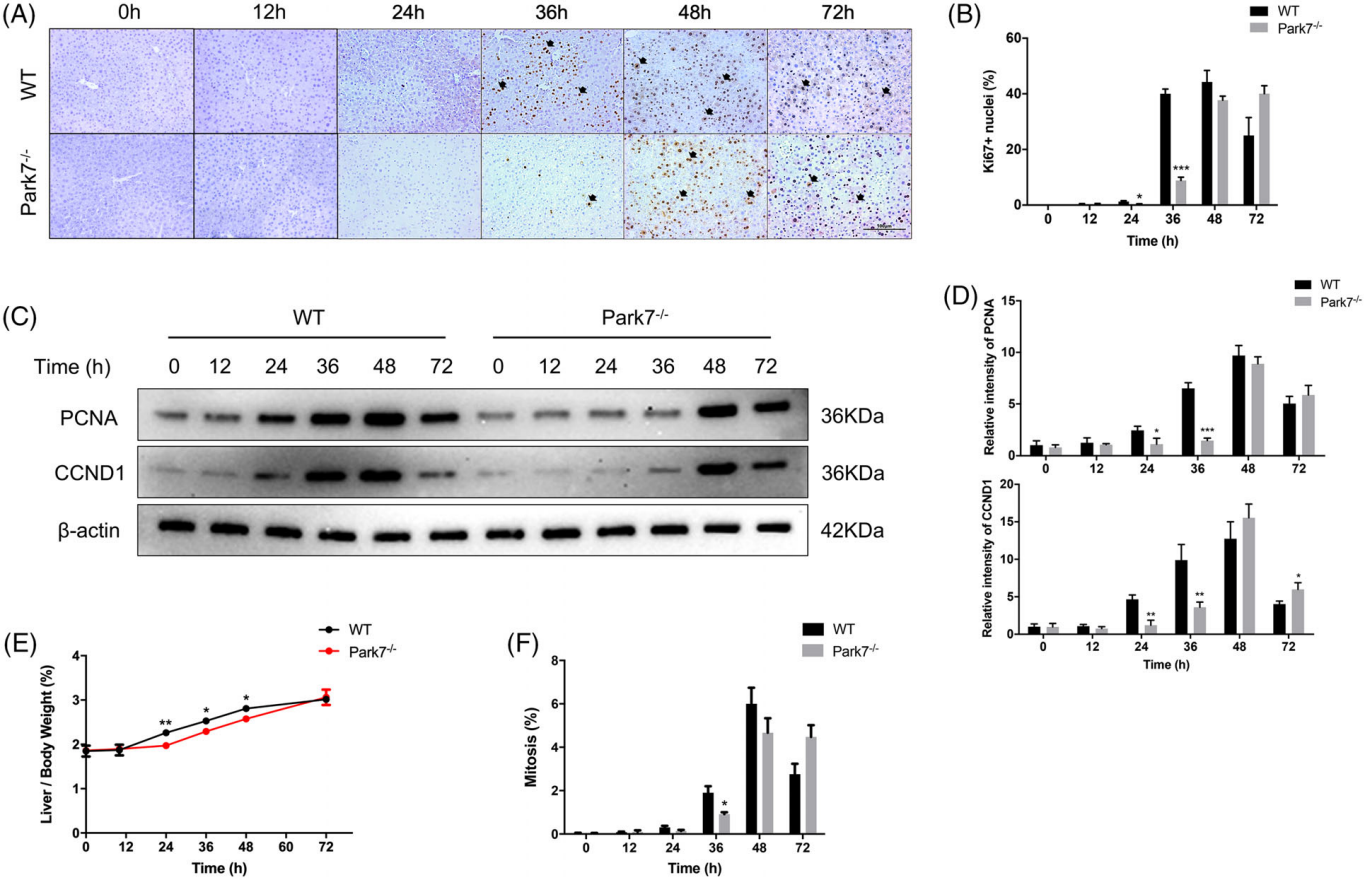
*Park7* deficiency delays liver regeneration post‐2/3 partial hepatectomy (PHx). Wild‐type and *Park7^−/−^* mice were subjected to PHx. (A) Immunohistochemistry of Ki67 at indicated time points after PHx was performed. (B) Percentages of Ki67 positive hepatocytes were calculated (*n* = 4–6 mice/group). (C) Immunoblot of PCNA/CCND1 at indicated time points after PHx was performed (*n* = 4–6 mice/group). β‐Actin was used as a loading control. (D) PCNA/CCND1 expression was quantified. Representative of three experiments. (E) Liver‐to‐body weight ratios were calculated at indicated time points (*n* = 4–6 mice/group). (F) Mitotic counts were calculated at indicated time points (*n* = 4–6 mice/group). Data are shown as mean ± SEM. *p < .05; **p < .01; ***p < .001. Scale bar: 100 μm

We performed 2/3 PHx in WT and *Park7^−/−^* mice and evaluated their liver regeneration potential by examining the Ki67 (a nuclear antigen in proliferating cells) and PCNA (a nuclear protein necessary for DNA synthesis)/CCND1 (cyclin D1; a marker of G1/S) levels and the liver‐to‐body weight ratios. As shown in Figure 1A,B, immunohistochemistry staining showed that the hepatic Ki67 levels peaked at 36–48 h in WT mice but at 48–72 h in *Park7^−/−^* mice after PHx. Consistently, western blot assays showed that compared with WT mice, *Park7^−/−^* mice displayed delayed PCNA and CCND1 induction at 24 and 36 h after PHx (Figure 1C,D). Moreover, significantly decreased liver‐to‐body weight ratios were observed in *Park7^−/−^* mice compared with WT mice at 24, 36 and 48 h after PHx (Figure 1E). Additionally, in agreement with the Ki67 results, lower and delayed cell mitotic counts were found at 36 h (Figure 1F). The induction of principal drivers of cell cycle progression was also delayed in *Park7^−/−^* mice compared with WT mice after PHx (Figure S2A–I), which revealed deficient S/M phase progression. These findings indicate that *Park7* deficiency significantly delays PHx‐induced liver regeneration.

### 2.3 Enhanced TRAS in the regenerating liver of *Park7* ^−/−^ mice after PHx

**FIGURE 2:**
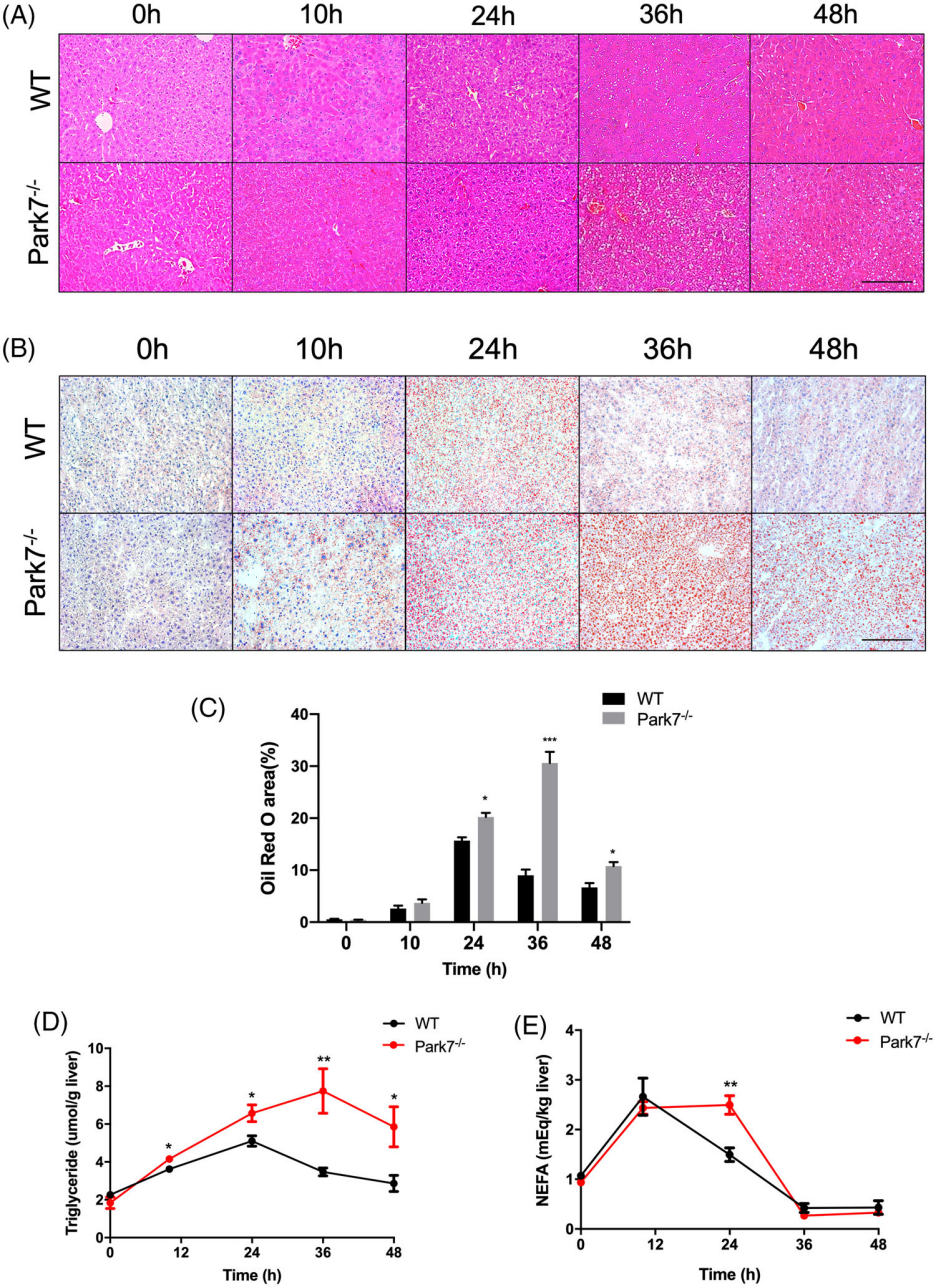
*Park7‐*deficient mice show enhanced transient regeneration–associated steatosis (TRAS) post‐2/3 partial hepatectomy (PHx). Wild‐type and *Park7^−/−^* mice were subjected to PHx. (A) Haematoxylin and eosin staining was performed at indicated time points after PHx. (B) Oil Red O staining was performed at indicated time points after PHx. (C) The quantitative analysis of Oil Red O staining according to the percentages of positive areas (*n* = 4–6 mice/group). (D) Hepatic triglycerides were evaluated at indicated time points after PHx (*n* = 4–6 mice/group). (E) Hepatic non‐esterified fatty acids were evaluated at indicated time points after PHx (*n* = 4–6 mice/group). Data are shown as mean ± SEM. *p < .05; **p < .01; ***p < .001. Scale bar: 100 μm

*Park7* is a key regulator of inflammatory responses.^1^ Given that inflammatory cytokines play an important role during the priming phase of liver regeneration,^30^, ^31^ we evaluated whether *Park7* affects this period in liver regeneration. Both the mRNA and serum levels of IL‐6 and TNFα were comparable in the priming phase between both genotypes (Figure S3A,B), suggesting that *Park7* did not affect the priming phase in the process of liver regeneration. Correspondingly, there was no difference in the hepatic activation of STAT3 in the priming phase of liver regeneration between WT and *Park7* ^−/−^ mice (Figure S3C,D). Interestingly, haematoxylin–eosin (HE) staining showed a greater number of fat droplets in the regenerating liver of *Park7^−/−^* mice compared with WT mice at 36 and 48 h after PHx (Figure 2A). Meanwhile, the difference in hepatic lipid accumulation between WT and *Park7^−/−^* mice was further explored by Oil Red O staining (Figure 2B,C). Additionally, ELISA assays showed that the transient hepatic accumulation of triglycerides (TG) and non‐esterified fatty acids (NEFA) was elevated in the regenerating liver of *Park7^−/−^* mice compared with WT mice (Figure 2D,E), along with liver cholesterol and cholesterol esters (Figure S4A,B). Interestingly, the serum NEFA level also increased in *Park7^−/−^* mice (Figure S5), which suggests enhanced adipose tissue–associated lipolysis in *Park7^−/−^* mice. *Park7* ^−/−^ mice also displayed a downregulation of peroxisome β‐oxidation genes, such as *Acox1* and *Crot*, at 24–48 or 36 h (Figure S6A,B). *Cd36*, *Fabp4*, *Fabp1* and *Fatp4* expression was similar between WT and *Park7* ^−/−^ mice, whereas *Fabp2* expression was decreased in *Park7* ^−/−^ mice during the regenerative phase, suggesting that *Park7* has little impact on lipid import (Figure S6C–G). Additionally, comparable mRNA levels of *Mttp* and *ApoB*‐100 showed similar VLDL assembly and excretion between the two groups (Figure S6H,I). These findings suggest that *Park7* deficiency does not affect the priming phase of hepatic regeneration but results in enhanced TRAS in regenerating liver.

### 2.4 Hepatocyte‐specific *Park7* deficiency is sufficient to enhance TRAS and delay liver regeneration after PHx

**FIGURE 3:**
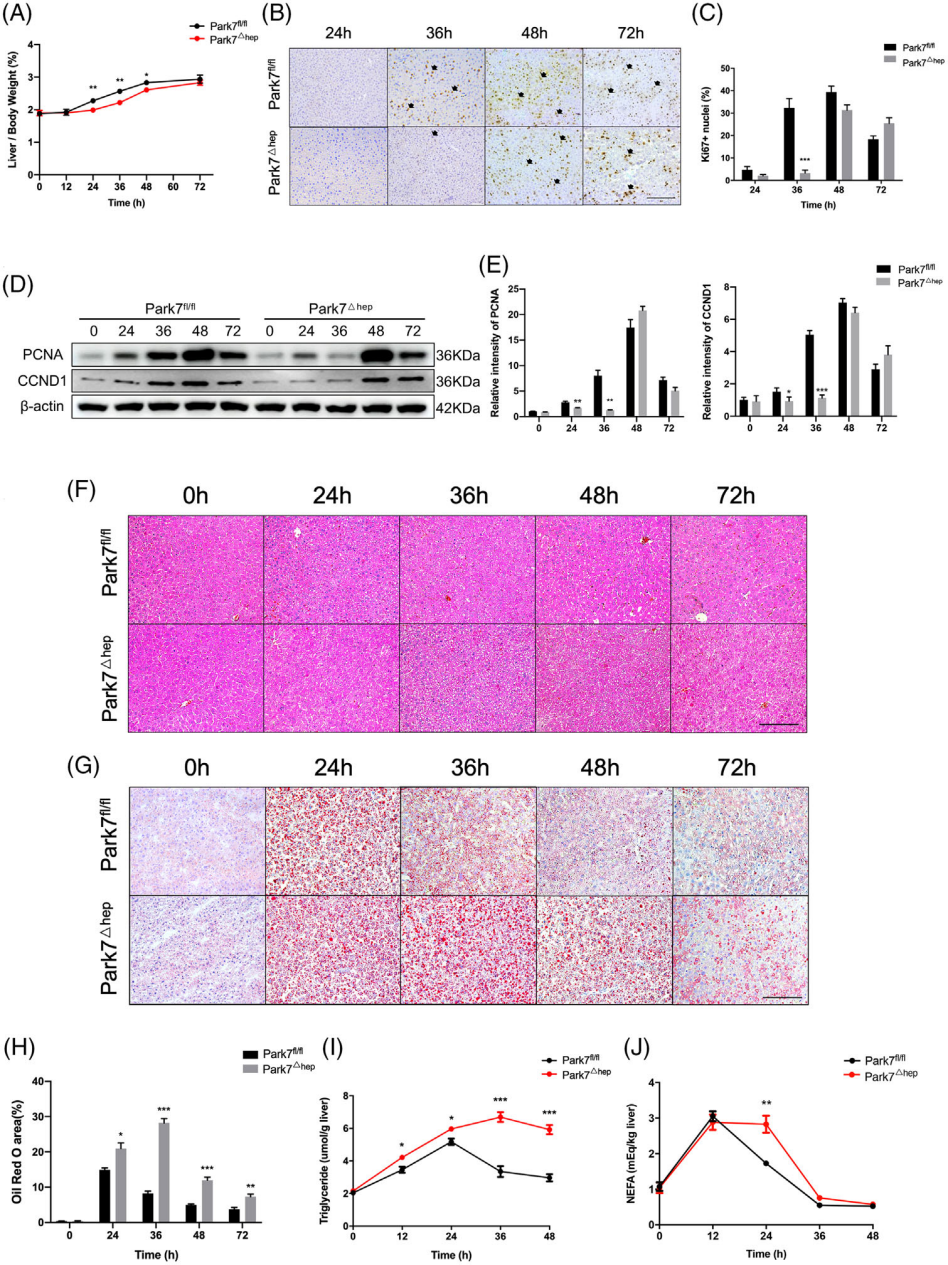
Hepatocyte‐specific *Park7* depletion is sufficient to delay liver regeneration and enhance transient regeneration–associated steatosis (TRAS) post‐2/3 partial hepatectomy (PHx). *Park7^fl/fl^* and *Park7^△hep^* mice were subjected to PHx. (A) Liver‐to‐body weight ratios were calculated at indicated time points after PHx (*n* = 4–6 mice/group). (B) Immunohistochemistry of Ki67 at indicated time points after PHx was performed. (C) Percentages of Ki67 positive hepatocytes were calculated (*n* = 4–6 mice/group). (D) Immunoblot of PCNA/CCND1 at indicated time points after PHx was performed (*n* = 4–6 mice/group). β‐Actin was used as a loading control. (E) PCNA/CCND1 expression was quantified. Representative of three experiments. (F) Haematoxylin and eosin (HE) staining was performed at indicated time points after PHx. (G) Oil Red O staining was performed at indicated time points after PHx. (H) The areas of positive staining were quantified (*n* = 4–6 mice/group). (I) Hepatic triglycerides were evaluated at indicated time points after PHx (*n* = 4–6 mice/group). (J) Hepatic non‐esterified fatty acids were evaluated at the indicated times after PHx (*n* = 4–6 mice/group). Data are shown as mean ± SEM. *p < .05; **p < .01; ***p < .001. Scale bar: 100 μm

To investigate whether hepatic *Park7* deficiency is sufficient to delay liver regeneration, we generated hepatocyte‐specific *Park7* knockout (*Park7* ^△hep^) mice by crossing *Park7* ^fl/fl^ mice with Alb‐Cre^+^ mice. As shown in Figure S7A,B, hepatic *Park7* deficiency was validated using a western blot assay. Compared with *Park7* ^fl/fl^ controls, *Park7* ^△hep^ mice showed severely impaired liver regeneration after PHx, as evidenced by decreased liver‐to‐body weight ratios (Figure 3A) and delayed Ki67 (Figure 3B,C) and PCNA/CCND1 induction (Figure 3D,E). Similar to the observations in *Park7* ^−/−^ mice, HE staining, Oil Red O staining and ELISA assays of TG and NEFA showed enhanced TRAS in the regenerating liver of *Park7* ^△hep^ mice compared with *Park7* ^fl/fl^ controls (Figure 3F–J). These findings indicate that hepatic *Park7* deficiency is sufficient to enhance TRAS and delay liver regeneration after PHx. Additionally, the blood glucose levels were elevated in *Park7* ^△hep^ mice at 24‐h post‐PHx, but the levels were comparable at other time points (Figure S8A). Liver glycogen was also investigated using biochemical assays and periodic acid–Schiff staining. We found that hepatocyte‐specific *Park7* deficiency further impaired glycogen storage (Figure S8B,C), which might result from mitochondrial dysfunction in *Park7* ^△hep^ mice after PHx.

### 2.5 *Park7* deficiency affects the expression of key regulators of β‐oxidation

**FIGURE 4:**
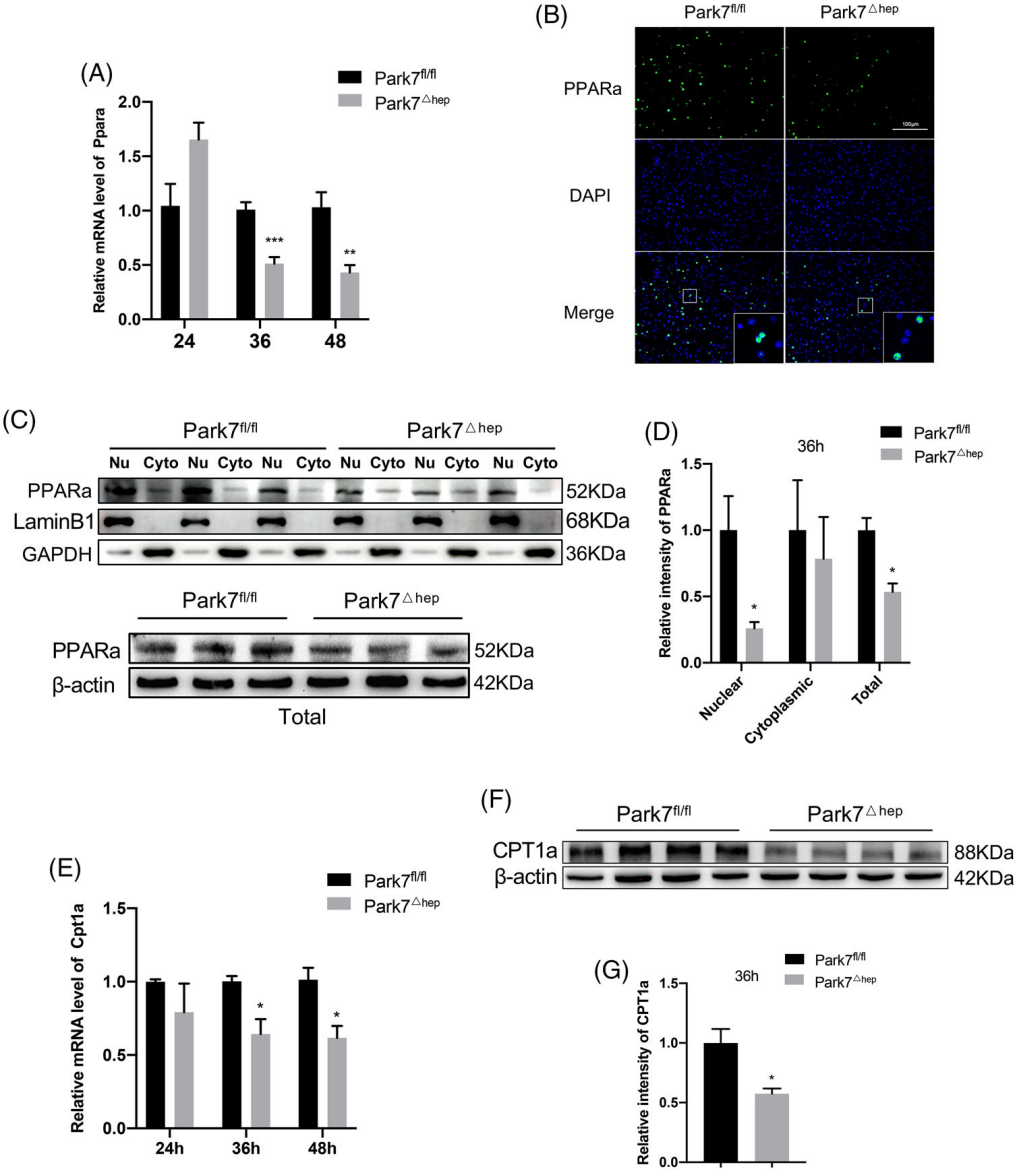
*Park7* deficiency suppresses hepatic PPARa and carnitine palmitoyltransferase 1a (CPT1a) expression post‐2/3 partial hepatectomy (PHx). *Park7^fl/fl^* and *Park7^△hep^* mice were subjected to PHx. (A) mRNA expression of *Ppara* at the indicated times post‐PHx was determined by quantitative polymerase chain reaction (qPCR) (*n* = 3–4 mice/group). (B) Immunofluorescent staining of PPARa at 36‐h post‐PHx. Nuclei were counterstained with DAPI. (C) Immunoblot of nuclear, cytosolic and total PPARa at 36 h after PHx (*n* = 4–6 mice/group). β‐Actin, LaminB1 and GAPDH were used as loading controls for total protein, a nuclear protein and cytoplasmic protein, respectively. (D) PPARa expression was quantified. Representative of three experiments. (E) mRNA expression of *CPT1a* at the indicated times post‐PHx was determined by qPCR (*n* = 3–4 mice/group). (F) Immunoblot of CPT1a at 36‐h post‐PHx was performed (*n* = 4–6 mice/group). β‐Actin was used as a loading control. (G) CPT1a expression was quantified. Representative of three experiments. Data are shown as mean ± SEM. *p < .05; **p < .01; ***p < .001. Scale bar: 100 μm

It is generally acknowledged that TRAS is a result of the uptake of adipose‐derived fat by the regenerating liver,^32^ which is a process unrelated to hepatic lipogenesis.^33^ In‐line with this notion, although the mRNA level of *Acc1* increased at 24 h after PHx and that of *Srebf1* decreased at 36 h, the mRNA levels of the main *Srebf1*‐targeted lipogenesis genes, such as *Fasn*, *Acc1* and *Scd1*, were comparable between *Park7* ^fl/fl^ and *Park7* ^△hep^ mice at 36 and 48‐h post‐PHx (Figure S9A). Similarly, despite an increasing level of ACC1 in *Park7* ^△hep^ mice, we found no significant differences between the protein levels of FASN, ACLY and SCD1 at 36 h after PHx, implying the inconspicuous effect of hepatic lipogenesis (Figure S9B,C). TRAS in the regenerating liver could be affected by fatty acid β‐oxidation. To investigate whether the enhanced TRAS in *Park7* ^△hep^ mice resulted from impaired β‐oxidation, we examined the level of *Ppara*, a key regulator of fatty acid β‐oxidation in liver tissues.^34^ Quantitative polymerase chain reaction (PCR) assays showed that hepatic *Ppara* was significantly downregulated in *Park7* ^△hep^ mice compared with *Park7* ^fl/fl^ controls at 36 and 48‐h post‐PHx (Figure 4A). Immunofluorescent (IF) staining and western blot assays of PPARa showed that either total PPARa levels or PPARa nuclear localization  was greatly reduced in *Park7* ^△hep^ mice at 36 h after PHx compared with *Park7* ^fl/fl^ controls (Figure 4B–D). As a result of decreased PPARa expression and nuclear localization, a decrease in the expression of *Cpt1a*, a downstream transcriptional target of PPARa and also a rate‐limiting enzyme of β‐oxidation, was found in *Park7* ^△hep^ mice at 36 and 48‐h post‐PHx (Figure 4E–G). We then examined the PPARa and CPT1a expression before and after PHx in detail. The PPARa and CPT1a expression was relatively low before PHx compared with the expression at 36 h after PHx, and the PPARa and CPT1a expression was slightly increased before PHx in *Park7* ^△hep^ mice (Figure S10A,B), suggesting that *Park7* plays a different role before and after PHx. Considering that PARK7 disruption delayed but ultimately did not prevent PHx‐induced hepatic regenerative recovery, we assumed that the drastic decrease in lipid accumulation after 48 h might stimulate liver regeneration. We therefore administered etomoxir, an inhibitor of CPT1a, every 12 h starting 36 h after PHx. The results showed that the liver‐to‐body weight ratios of *Park7* ^△hep^ mice started to become comparable with *Park7* ^fl/fl^ mice from 60 h after PHx in phosphate‐buffered saline (PBS)‐treated group, whereas the ratios of *Park7* ^△hep^ mice failed to catch up with *Park7* ^fl/fl^ mice in etomoxir‐treated groups at 60–96 h after PHx. These results indicated that the FAO levels started to increase after 48 h to promote liver regeneration by supplying greater amounts of energy (Figure S11). To sum up, these findings suggest that hepatocyte‐specific *Park7* deficiency negatively affects fatty acid β‐oxidation in the proliferation phase of liver regeneration,^35^ which in turn results in enhanced TRAS and delayed liver regeneration.

### 2.6 PTEN deficiency rescues delayed liver regeneration in *Park7* ^△hep^ mice

**FIGURE 5:**
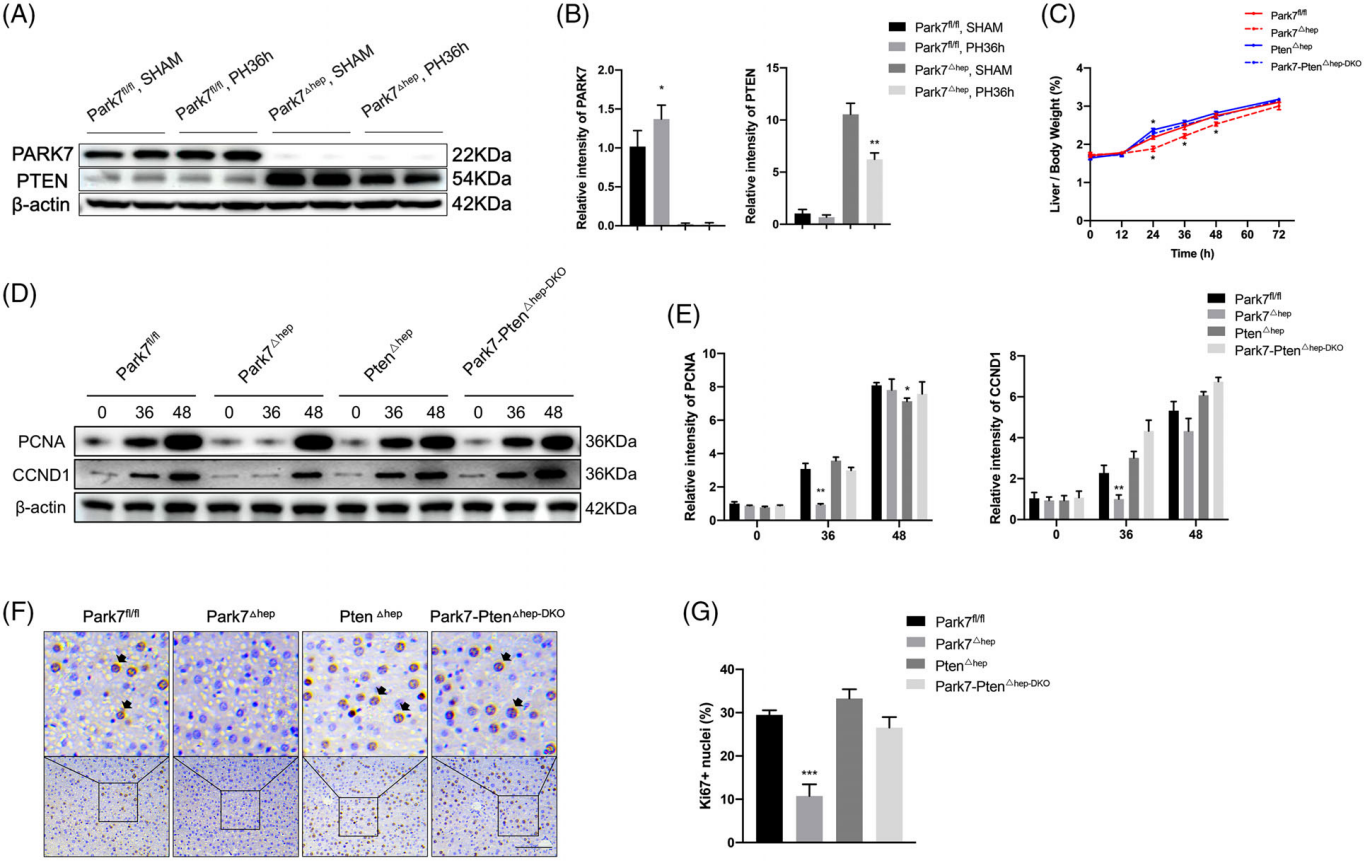
*Pten* deficiency rescues the delayed liver regeneration post‐2/3 partial hepatectomy (PHx) in *Park7* ^△hep^ mice. *Park7^fl/fl^*, *Park7^△hep^*, *Pten^△hep^* and *Park7‐Pten* ^△hep‐DKO^ mice were subjected to PHx. (A) Immunoblot of hepatic PARK7 and phosphatase and tensin homolog (PTEN) before or after PHx was performed (*n* = 4–6 mice/group). β‐Actin was used as a loading control. (B) PARK7 and PTEN expression was quantified. Representative of three experiments. (C) Liver‐to‐body weight ratios were calculated at indicated time points after PHx (*n* = 4–6 mice/group). (D) Immunoblot of PCNA/CCND1 at indicated time points after PHx was performed (*n* = 4–6 mice/group). β‐Actin was used as a loading control. (E) PCNA/CCND1 expression was quantified. Representative of three experiments. (F) Immunohistochemistry of hepatic Ki67 signals at 36 h after PHx was performed. (G) Percentages of Ki67 positive hepatocytes were calculated (*n* = 4–6 mice/group). Data are shown as mean ± SEM. *p < .05; **p < .01; ***p < .001. Scale bar: 100 μm

*Park7* is a negative regulator of PTEN. Although several previous studies have demonstrated that *Park7* exclusively inhibits the phosphatase activity of PTEN instead of suppressing PTEN expression,^28^, ^36^ we detected remarkable PTEN induction in the liver tissues with *Park7* deficiency (Figure 5A,B), suggesting that *Park7* can negatively regulate PTEN expression. Given that PTEN negatively regulates liver regeneration, we hypothesized that the delayed liver regeneration in *Park7* ^△hep^ mice is caused by enhanced PTEN expression. To this end, we generated hepatocyte‐specific *Park7‐Pten* double knockout (*Park7‐Pten* ^△hep‐DKO^) mice and confirmed PARK7 and PTEN deficiency in liver tissues using western blot assays (Figure S12A,B). Compared with *Park7* ^fl/fl^ controls, significantly decreased liver‐to‐body weight ratios (Figure 5C) and hepatic PCNA/CCND1 and Ki67 levels (Figure 5D–G) were only observed in *Park7* ^△hep^ mice but not in *Park7‐Pten* ^△hep‐DKO^ mice at indicated time points post‐PHx, suggesting that PTEN may be attributed to the delayed liver regeneration in *Park7* ^△hep^ mice. An increase in the liver‐to‐body weight ratio at 24‐h post‐PHx was observed in *Pten* ^△hep^ mice compared with *Park7* ^fl/fl^ controls (Figure 5C), which is consistent with a previous finding revealing that PTEN deficiency enhanced liver regeneration.^6^ Meanwhile, the delayed induction of pro‐cell cycle factors in *Park7* ^△hep^ mice was restored in *Park7‐Pten* ^△hep‐DKO^ mice after PHx (Figure S12C–H). Additionally, we estimated the liver‐to‐body weight ratios before PHx. We found no statistically significant differences between these four groups at baseline (Figure S13A). Moreover, comparable liver‐to‐body weight ratios were observed in *Park7* ^fl/fl^, *Park7* ^△hep^ and *Park7‐Pten* ^△hep‐DKO^ mice, whereas *Pten* ^△hep^ mice displayed an increased liver‐to‐body weight ratio at 168 h after the completion of the regenerative response to PHx (Figure S13B). Collectively, these data indicate that PTEN is required for delayed liver regeneration in *Park7* ^△hep^ mice and that PTEN deficiency can restore impaired liver regeneration in *Park7* ^△hep^ mice.

### 2.7 Functional inhibition of PTEN is sufficient to rescue delayed liver regeneration in *Park7* ^△hep^ mice

**FIGURE 6:**
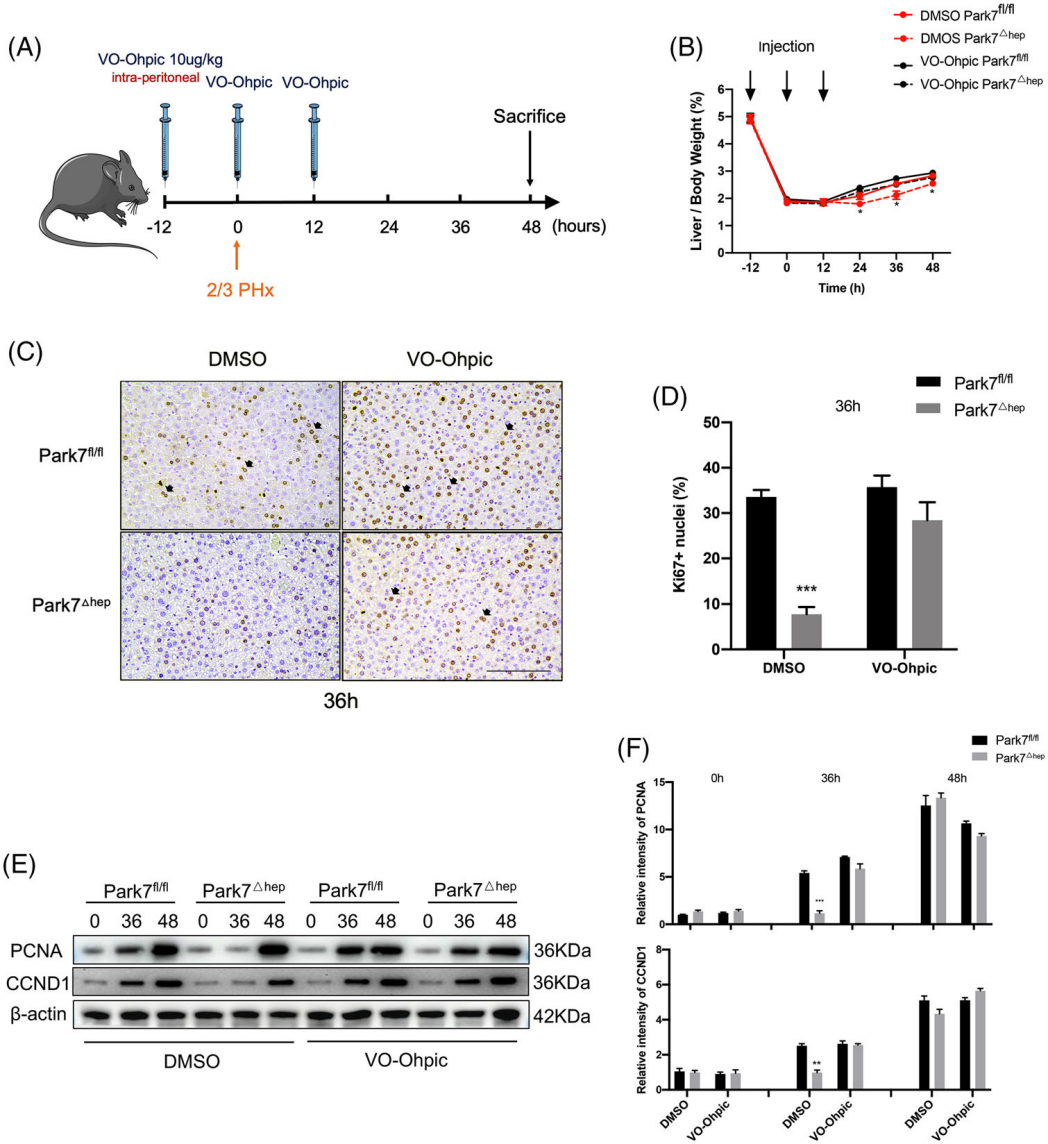
Phosphatase and tensin homolog (PTEN) inhibition rescues the delayed liver regeneration post‐2/3 partial hepatectomy (PHx) in *Park7^△hep^* mice. *Park7^fl/fl^* and *Park7^△hep^* mice were subjected to PHx. (A) The VO‐Ohpic treatment regimen is graphically illustrated. (B) Liver‐to‐body weight ratios were calculated at indicated time points after PHx (*n* = 4–6 mice/group). (C) Immunohistochemistry of hepatic Ki67 at 36 h after PHx was performed. (D) Percentages of Ki67 positive hepatocytes were calculated (*n* = 4–6 mice/group). (E) Immunoblot of PCNA/CCND1 at indicated time points post‐PHx was performed (*n* = 4–6 mice/group). β‐Actin was used as a loading control. (F) PCNA/CCND1 expression was quantified. Representative of three experiments. Data are shown as mean ± SEM. *p < .05; **p < .01; ***p < .001. Scale bar: 100 μm

As a phosphatase, PTEN executes its function via dephosphorylation of downstream targets. To further examine the involvement of PTEN in the delayed liver regeneration in *Park7* ^△hep^ mice, we treated *Park7* ^fl/fl^ and *Park7* ^△hep^ mice with VO‐Ohpic,^2^ a PTEN inhibitor, followed by a PHx‐induced liver regeneration model. The treatment regimen is graphically illustrated in Figure 6A. The administration of VO‐Ohpic diminished the differences in the liver‐to‐body weight ratios (Figure 6B) and hepatic Ki67 (Figure 6C,D) and PCNA/CCND1 (Figure 6E,F) levels between *Park7* ^fl/fl^ and *Park7* ^△hep^ mice. These findings suggest that inhibiting the phosphatase activity of PTEN is sufficient to rescue the delayed liver regeneration in *Park7* ^△hep^ mice after PHx.

### 2.8 PTEN deficiency or inhibition restores PPARa and CPT1a expression and hepatic lipid utilization in the regenerating liver of *Park7* ^△hep^ mice

**FIGURE 7:**
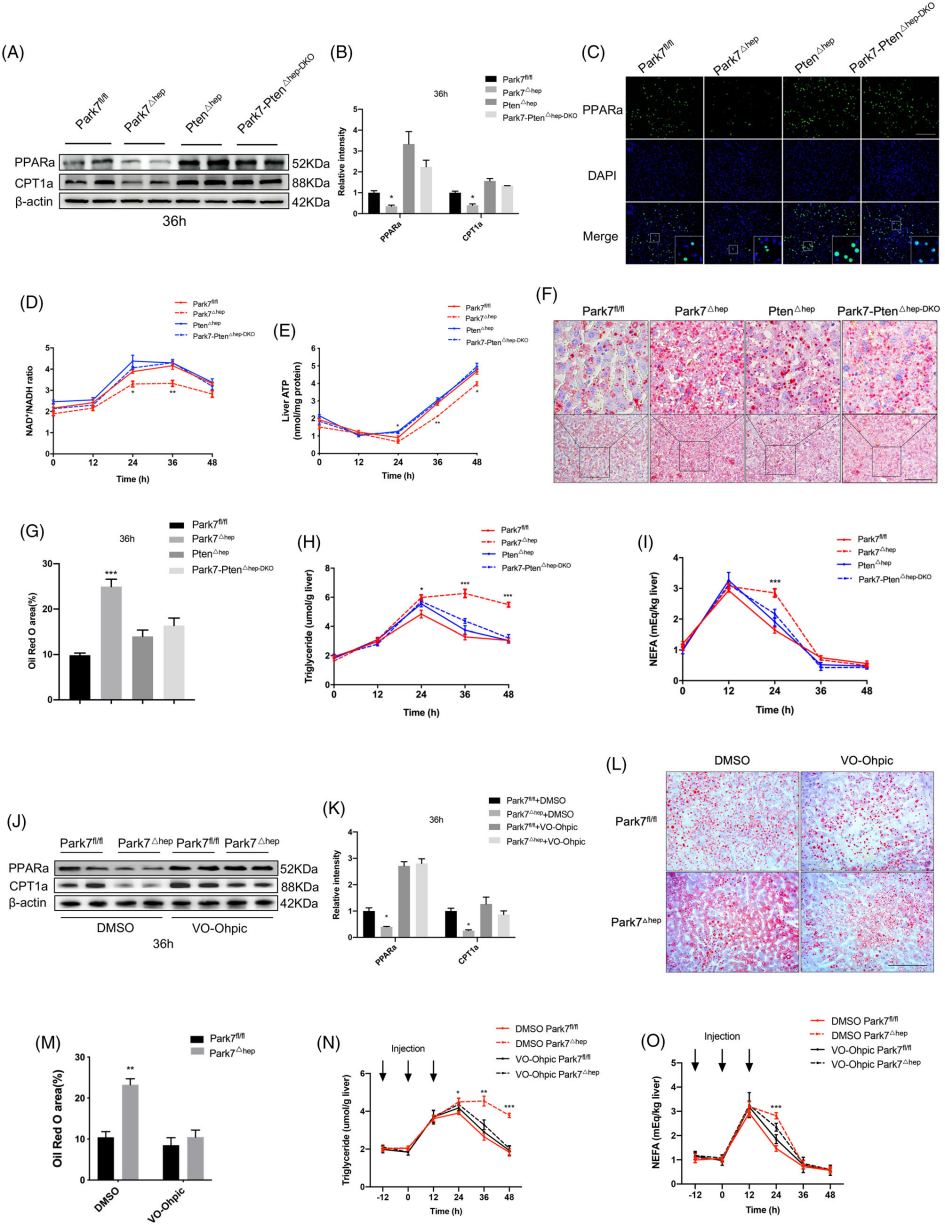
*Pten* deficiency and inhibition enhance β‐oxidation and decrease transient regeneration–associated steatosis (TRAS) post‐2/3 partial hepatectomy (PHx). Parkinsonism‐associated deglycase *(Park7)^fl/fl^*, *Park7^△hep^*, *Pten^△hep^* and *Park7‐Pten* ^△hep‐DKO^ mice were subjected to PHx. (A) Immunoblot of hepatic peroxisome proliferator‐activated receptor‐a (PPARa) and carnitine palmitoyltransferase 1a (CPT1a) at 36‐h post‐PHx was performed (*n* = 4–6 mice/group). β‐Actin was used as a loading control. (B) PPARa and CPT1a expression was quantified. Representative of three experiments. (C) Immunofluorescent staining of hepatic PPARa at 36‐h post‐PHx. Nuclei were counterstained with DAPI. (D) Biochemical detection of intracellular NAD^+^/NADH ratios in *Park7^fl/fl^*, *Park7^△hep^*, *Pten^△hep^* and *Park7‐Pten* ^△hep‐DKO^ mice at indicated times after PHx (*n* = 3–4 mice/group). (E) Biochemical detection of hepatic ATP levels at the indicated times post‐2/3 PHx (*n* = 3–4 mice/group). (F) Oil Red O staining of hepatic lipid accumulation was performed at 36‐h post‐PHx. (G) The areas of positive staining were quantified (*n* = 4–6 mice/group). (H and I) Hepatic triglycerides (TG) (H) and non‐esterified fatty acids (NEFA) (I) in *Park7^fl/fl^*, *Park7^△hep^*, *Pten^△hep^*, *Park7‐Pten* ^△hep‐DKO^ mice were measured at indicated time points after PHx (*n* = 3–4 mice/group). (J) Immunoblot of hepatic PPARa and CPT1a at 36 h after PHx was performed in *Park7^fl/fl^* and *Park7^△hep^* mice with or without VO‐Ohpic treatment (*n* = 4–6 mice/group). β‐Actin was used as a loading control. (K) PPARa and CPT1a expression were quantified. Representative of three experiments. (L) Oil Red O staining of liver tissues was performed at 36 h after PHx in *Park7^fl/fl^* and *Park7^△hep^* mice with or without VO‐Ohpic treatment. (M) The areas of positive staining were quantified (*n* = 4–6 mice/group). (N and O) Hepatic TG (N) and NEFA (O) were measured at indicated time points after PHx in *Park7^fl/fl^* and *Park7^△hep^* mice with or without VO‐Ohpic treatment (*n* = 3–4 mice/group). Data are shown as mean ± SEM. *p < .05; **p < .01; *** *p* < .001. Scale bar: 100 μm

To investigate whether PTEN deficiency or inhibition restores the β‐oxidation impairment in the regenerating liver of *Park7* ^△hep^ mice, we first compared the changes in hepatic expression of PPARa and CPT1a between *Park7* ^△hep^ and *Park7‐Pten* ^△hep‐DKO^ mice after PHx. Compared with *Park7* ^fl/fl^ controls, western blot assays showed a decreased hepatic expression of PPARa and CPT1a in *Park7* ^△hep^ mice and restored the expression of these two genes in *Park7‐Pten* ^△hep‐DKO^ mice at 36 h after PHx (Figure 7A,B). IF staining showed reduced PPARa nuclear localization in the regenerating liver of *Park7* ^△hep^ mice and increased nuclear PPARa signals in the regenerating liver of *Park7‐Pten* ^△hep‐DKO^ mice at 36‐h post‐PHx (Figure 7C). The mRNA levels of PPARa target genes, such as *Acox1*, *Crot* and *Hmgcs2*, and other β‐oxidation‐associated genes, such as *Acadm* and *Slc25a20*, were also examined and validated the changes in PPARa (Figure S14A–E). To further confirm whether *Park7* affects the rate of β‐oxidation, we examined the NAD^+^/NADH ratio levels. Supporting our conclusion, *Park7* deletion decreased the NAD^+^/NADH ratio 24–36 h after PHx, which was restored in *Park7‐Pten* ^△hep‐DKO^ mice (Figure 7D). Additionally, compared with *Park7* ^fl/fl^ controls, *Park7* ^△hep^ mice displayed decreased serum β‐hydroxybutyrate levels (Figure S15A). We next measured the ketogenesis in vivo. *Park7* ^△hep^ mice displayed severe impairment in their ability to produce β‐hydroxybutyrate compared with *Park7* ^fl/fl^ controls, which was also restored in *Park7‐Pten* ^△hep‐DKO^ mice (Figure S15B). We then found that hepatic malonyl CoA levels increased in *Park7* ^△hep^ mice and became comparable between *Pten* ^△hep^ and *Park7‐Pten* ^△hep‐DKO^ mice (Figure S15C). The previous findings suggest that PTEN deficiency restores the impaired β‐oxidation in *Park7* ^△hep^ mice. Consequently, the regenerating liver of *Park7‐Pten* ^△hep‐DKO^ mice produced significantly high levels of ATP compared with that of *Park7* ^△hep^ mice (Figure 7E). As a result of the enhanced β‐oxidation, Oil Red O staining of hepatic lipids and ELISA assays of hepatic TG and NEFA levels showed reduced lipid accumulation in the regenerating liver of *Park7‐Pten* ^△hep‐DKO^ mice at 36 h after PHx (Figure 7F–I). Consistent with the results of genomic *Pten* deficiency, the inhibition of the phosphatase activity of PTEN by VO‐Ohpic significantly restored the expression of PPARa and CPT1a in *Park7* ^△hep^ mice at 36‐h post‐PHx (Figure 7J,K). Meanwhile, the results of Oil Red O staining and assays of hepatic lipids showed that the difference in TRAS after PHx between *Park7* ^fl/fl^ and *Park7* ^△hep^ mice was diminished upon VO‐Ohpic treatment (Figure 7L–O). Therefore, these findings suggest that PTEN deficiency or inhibition reverses the β‐oxidation impairment in *Park7* ^△hep^ mice and results in accelerated utilization of hepatic lipids derived from TRAS.

### 2.9 Hepatocyte growth factor (HGF)/epidermal growth factor (EGF)‐activated NRF2 plays a key role in regulating *Park7* expression after PHx

**FIGURE 8:**
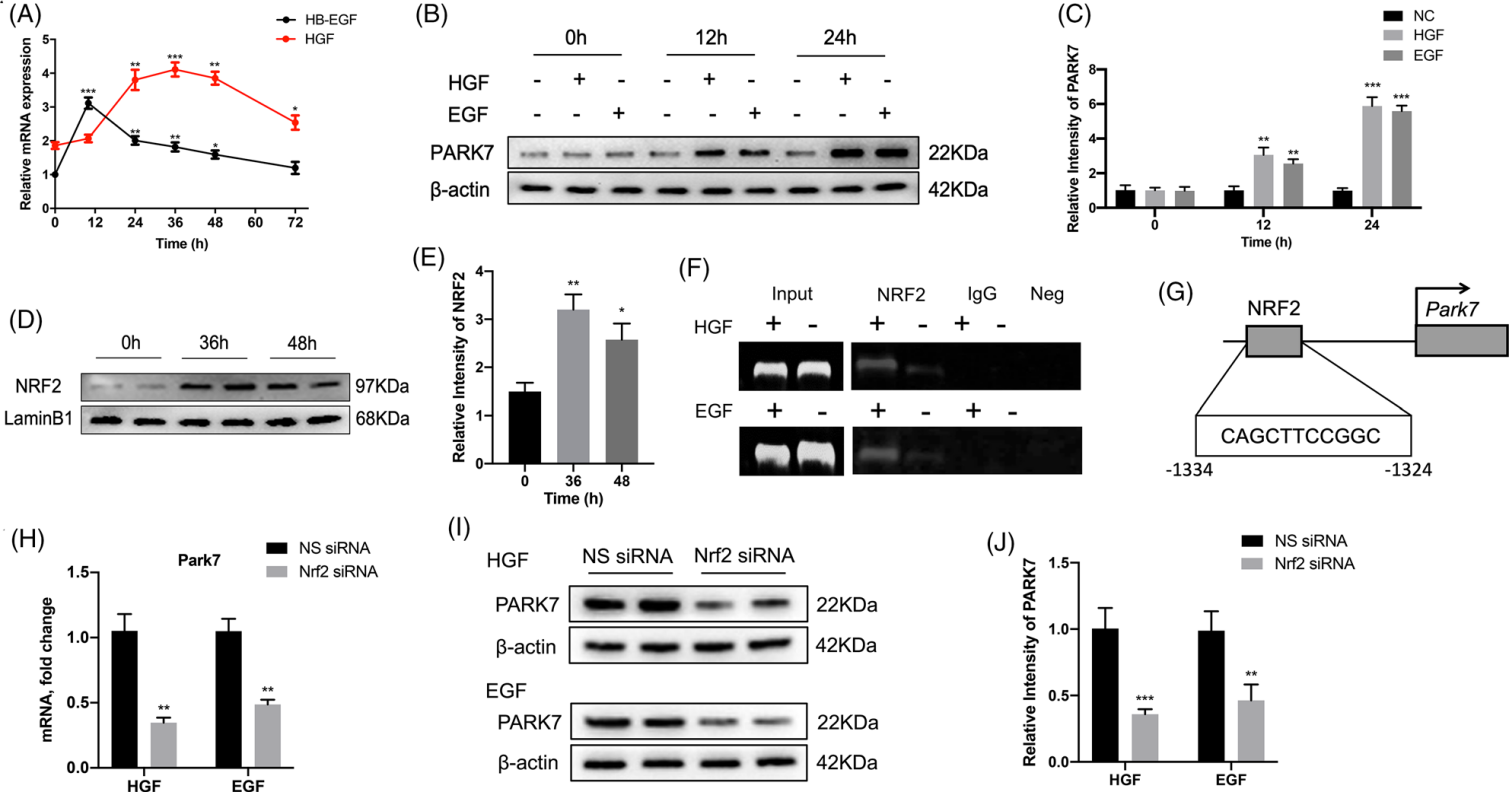
Hepatocyte growth factor (HGF)/epidermal growth factor (EGF)‐activated erythroid 2–related factor 2 (NRF2) plays a key role in regulating *Park7* expression post–partial hepatectomy (PHx). (A) mRNA levels of HGF and epidermal growth factor (HB)‐EGF at the indicated times post‐PHx were determined by quantitative polymerase chain reaction (qPCR) (*n* = 3–4 mice/group). (B) Immunoblot of PARK7 in AML12 cells incubated with HGF or EGF (50 ng/ml) for 12 and 24 h was performed (*n* = 3 replicates/group). β‐Actin was used as a loading control. (C) PARK7 expression was quantified. Representative of three experiments. (D) Immunoblot of nuclear NRF2 at 36 and 48‐h post‐PHx was performed (*n* = 4–6 mice/group). LambinB1 was used as a loading control. (E) NRF2 expression was quantified. Representative of three experiments. (F) ChIP–PCR analysis of NRF2 binding to the *Park7* promoter. Protein‐bound chromatin was prepared from AML12 and immunoprecipitated with NRF2 antibody, and then the immunoprecipitated DNA was analysed by PCR. Neg, negative control without DNA template. (G) Localization of NRF2‐binding sites on mouse *Park7*. (H) mRNA expression of *Park7* in AML12 cells treated with HGF or EGF (50 ng/ml) for 24 h between NS‐siRNA and *Nrf2*‐siRNA groups (*n* = 3 replicates/group). (I) Immunoblot of PARK7 in AML12 cells treated with HGF or EGF between NS‐siRNA and *Nrf2*‐siRNA groups (*n* = 3 replicates/group). β‐Actin was used as a loading control. (J) PARK7 expression was quantified. Representative of three experiments. Data are shown as mean ± SEM. *p < .05; **p < .01; *** *p* < .001

To further investigate the upstream regulatory factor of *Park7*, we first detected the mRNA levels of hepatocyte growth factor (HGF) and heparin‐binding epidermal growth factor‐like growth factor (HB‐EGF) in the liver at indicated time points after PHx (Figure 8A). We found that the mRNA levels of HGF peaked at 24–48 h after PHx and those of HB‐EGF peaked at 12 h and were maintained at a relatively high level at 12–48 h after PHx. To further confirm whether HGF/EGF upregulates *Park7* expression, AML12 cells were treated with HGF or EGF (50 ng/ml) for 12 and 24 h. PARK7 was upregulated in AML12 cells after HGF and EGF treatment (Figure 8B,C), suggesting that HGF/EGF induces *Park7* expression. Previously, it has been reported that HGF and EGF can induce the activation of NRF2, an important transcription factor associated with oxidative stress.^37^ Next, we found that the NRF2 level in the nucleus was markedly elevated at 36 and 48‐h post‐PHx (Figure 8D,E). Using PROMO, a virtual laboratory for the identification of putative transcription factor binding sites in DNA sequences from a species or groups of species of interest, we found that the promoter region of *Park7* carries a putative NRF2 binding site. Next, a ChIP–PCR assay was performed using NRF2 antibodies in HGF or EGF (50 ng/ml)‐stimulated AML12 cells. The ChIP assay showed increased NRF2 recruitment to the promoter region of *Park7* upon HGF or EGF treatment (Figure 8F,G). To further confirm this, we performed an *Nrf2* knockdown experiment using *Nrf2* siRNA. The results displayed that *Nrf2* knockdown decreased both mRNA and protein levels of PARK7 in AML12 cells treated with HGF or EGF (Figure 8H–J). These results reveal that *Park7* is a target gene of NRF2 in the regenerating liver.

### 2.10 oxPARK7 is responsible for PARK7‐mediated regulation of liver regeneration and hepatic β‐oxidation

We and other groups have shown that PARK7 is involved in the pathogenesis of several liver diseases based on its redox function. As reported previously,^38^ the Cys106 of PARK7 is oxidized under oxidative stress and is critical for the biological function of PARK7. We detected the levels of liver malondialdehyde (MDA) and 2′,7′‐dichlorofluorescin diacetate to analyse the oxidative status. The MDA levels were upregulated in *Park7* ^fl/fl^ mice at 12–48 h after PHx, as reported previously.^39^ Additionally, compared with *Park7* ^fl/fl^ mice, the MDA and ROS levels were elevated in *Park7* ^△hep^ mice at 36 and 48‐h post‐PHx (Figure S16A,B), which suggests that PARK7 also functions as an antioxidant response regulator in our study. We then examined the oxPARK7 expression in WT mice after PHx. Interestingly, elevated hepatic oxPARK7 expression was detected at 24–48 h after PHx (Figure S17A,B).

To investigate whether the role of PARK7 in PHx relies on its oxidized form, we established a hepatic overexpression of PARK7^WT^ or PARK7^C106A^ in *Park7* ^△hep^ mice by the administration of AAV‐PARK7^WT^ or AAV‐PARK7^C106A^ by tail vein injection. After AAV administration, the mice were fed with a normal diet for another 6 weeks and were then subjected to 2/3 PHx and sacrificed after 36 h. By examining the PCNA/CCND1 and Ki67 levels at 36 h after PHx, we found that the hepatic administration of PARK7^WT^, but not PARK7^C106A^, could rescue the impaired liver regeneration in *Park7* ^△hep^ mice (Figure S17C–F). Consistently, Oil Red O staining and TG measurement revealed that the hepatic expression of PARK7^WT^, but not PARK7^C106A^, could restore hepatic lipid utilization compared with *Park7* ^△hep^ mice after PHx (Figure S17G–I). The hepatic expression of PARK7^WT^, but not PARK7^C106A^, restored the hepatic expression of PPARa and CPT1a at 36 h after PHx (Figure S17J,K). Next, we examined serum ketone and liver ATP levels. Hepatic PARK7^WT^ overexpression resulted in increased serum β‐hydroxybutyrate and liver ATP levels compared with hepatic PARK7^C106A^ expression at 36‐h post‐PHx (Figure S17L,M). These results indicate that the PARK7 function in liver regeneration and hepatic β‐oxidation was dependent on its antioxidant capacity.

## 3 DISCUSSION

In the present study, we investigated the role of *Park7* in liver regeneration after major hepatectomy. We first showed that the hepatocyte‐specific depletion of *Park7* significantly increased TRAS and retarded liver regeneration post‐PHx. *Park7* deficiency suppressed *Ppara* and *Cpt1a* expression, resulting in the decreased β‐oxidation of fatty acids and ATP production in the regenerating liver. This was caused by relieving the *Park7*‐mediated inhibition of PTEN, as either the genomic depletion or functional inhibition of PTEN could reverse the downregulation of *Ppara* and *Cpt1a* and the reduction of β‐oxidation and ATP production in the regenerating liver of the *Park7* knockout mice. Therefore, by modulating PTEN expression and function, *Park7* could affect fatty acid oxidation and ATP production and act as a therapeutic target for liver regeneration.

Liver regeneration post‐PHx is a multi‐level process consisting of priming, proliferation and termination.^35^ The proliferation phase lasts for 48 h. In this period, hepatocytes progress to the S phase of the cell cycle and synthesize DNA, followed by substantial proliferation.^40^ The priming phase of liver regeneration has been widely investigated; however, the underlying mechanism has not been completely elucidated. The present study revealed a critical role of *Park7* in the proliferation phase of liver regeneration.

PARK7/DJ1 is a multifunctional protein and plays a critical role in cell death and cellular protection by acting as a redox‐sensitive chaperone and oxidative stress sensor.^41^ Many researchers, including us, have shown that *Park7* is involved in the process of many liver diseases. The emerging roles of *Park7* in glucose homeostasis and energy expenditure have been reported previously.^42^, ^43^, ^44^, ^45^ *Park7* expression increases in pancreatic islets of an aged mouse or human, protecting the mitochondrial integrity and avoiding the development of glucose intolerance and reduced β‐cell area.^43^ Mice with *Park7* deficiency showed resistance to high‐fat diet (HFD)‐induced obesity by enhancing energy expenditure.^45^ We previously found that *Park7* ^−/−^ mice were resistant to HFD‐related hepatic steatosis by expediting free fatty acid (FFA) utilization.^44^ This is consistent with a previous finding showing that hepatic PTEN overexpression ameliorated the development of hepatic steatosis in liver‐specific HuR knockout mice,^46^ given that *Park7* is a negative regulator of PTEN. In contrast to the reduced diet‐induced hepatic steatosis in *Park7^−^* ^/−^ mice, we found that *Park7* deficiency increased TRAS, suggesting that the underlying mechanisms of diet‐induced hepatic steatosis and TRAS are different. Indeed, unlike pathological steatosis, TRAS is a physiological process observed in each regenerating liver.^3^ In this study, as TRAS gradually reduced to lean values 48–72 h after the proliferation phase, the dependence of liver regeneration on TRAS gradually decreased. *Park7* started to function as an inhibitor of FAO, and the level of β‐oxidation in the *Park7* ^△hep^ mice gradually overtook the level in the *Park7* ^fl/fl^ mice (Figure S11). As a result, increasing Ki67, PCNA, CCND1 and mitotic count levels were observed from 48 to 72‐h post‐PHx (Figure 1), and *Park7* deficiency ultimately did not decrease liver/body ratios. To summarize, *Park7* deletion delayed and did not inhibit TRAS utilization, revealing a critical role of *Park7* in the proliferation phase of liver regeneration.

PTEN functions as the most critical inhibitor of the PI3K pathway. Surprisingly, both PTEN inhibition and expression inhibit β‐oxidation, most likely via two different mechanisms; one mechanism is dependent on the PI3K/AKT pathway, and the other is independent.^47^, ^48^ The mechanism of the latter remains unknown. On the one hand, functions associating with PTEN nuclear localization might explain the underlying mechanism. On the other hand, the phosphatase‐independent role of PTEN in regulating non‐canonical signalling pathways, such as the eukaryotic initiation factor 2α phosphorylation cascade, the c‐Jun N‐terminal kinase signalling pathway and MSP58‐mediated cellular transformation,^48^ might be involved in the function of PTEN‐related lipid catabolism stimulation. The factors inducing PTEN to function as either an inhibitor or a stimulator of lipid catabolism need to be further investigated in the future. Compared with the quiescent liver, PTEN disruption leads to a different result in the 2/3 PHx liver, where fat is a preferred energy source to its regeneration. The most remarkable function of PTEN is to counteract the activity of PI3K. These kinases regulate signals mediated by insulin‐like growth factors, insulin, and many other molecules involved in cellular metabolism, growth, proliferation and survival.^49^ To maintain euglycaemia stably and improve insulin resistance after hepatectomy, the PI3K signalling pathway is activated and plays a unique role in regenerating the liver. Therefore, PTEN‐mediated β‐oxidation alteration relies on the PI3K signalling pathway.

PARK7 has three cysteine residues located at positions 46, 53 and 106. The cysteine 106 residue is vital for the antioxidant activity of PARK7. Using adeno‐associated viruses expressing either PARK7^WT^ or PARK7^C106A^ mutant, we successfully proved that the function of PARK7 in the PARK7‐PTEN regulatory axis depended on the C106 cysteine residue. In addition, PARK7 acts as a regulator and stabilizer of NRF2, a redox‐sensitive transcription factor, which is a primary regulator of antioxidant genes.^50^ As previously reported, *Park7* activates the ERK1/2 pathway by inhibiting DUSP1 expression. In turn, oxidative stress can upregulate *Park7* by activating the ERK1/2 pathway, which acts as a positive feedback loop.^51^ Interestingly, the ChIP–PCR analysis showed that *Park7* could also act as a target gene of NRF2, which might also generate a positive feedback loop of oxidative stress.^52^

In this study, we showed that the increased TRAS and delayed liver regeneration in the *Park7* ^−/−^ mice were attributed to hepatic PTEN overexpression, which in turn inhibited FFA oxidation. This was confirmed by using VO‐Ohpic, a PTEN inhibitor, and *Park7‐Pten* ^△hep‐DKO^ mice, as either VO‐Ohpic treatment or *Pten* knockout reversed the increased TRAS and delayed liver regeneration in the *Park7* ^−/−^ mice, suggesting that PTEN functions as a predominant bridge between *Park7* and FFA‐β‐oxidation. Given that the PARK7‐PTEN regulatory axis is largely involved in the progression of many cancers,^26^, ^27^, ^28^ and FFA‐β‐oxidation is essential for cancer cell proliferation, survival, stemness and drug resistance,^53^ *Park7* may also serve as a potential therapeutic target for cancers with functional *Pten* expression.

## 4 MATERIALS AND METHODS

### 4.1 Human samples

Serum samples of seven patients were collected before and after PHx at 24 and 72 h from the left lateral lobectomy of normal donors who donated parts of the healthy livers to their children receiving paediatric living donor liver transplant at the Department of Liver Surgery, Renji Hospital, School of Medicine, Shanghai Jiao Tong University. Blood samples were incubated for 2 h at 37°C, centrifuged at 3000 rpm for 15 min at 4°C, and the supernatant was then collected. Matched pairs of serum and plasma were collected under the approval of the Institutional Ethic Committee of Renji Hospital of Shanghai Jiao Tong University. Written informed consent was obtained from each patient based on the policies of the committee. All research was performed in accordance with the government policies and the Declaration of Helsinki.

### 4.2 Animals

*Park7^−/−^* mice were obtained from the Jackson Laboratory (Bar Harbor, Maine, US), and C57BL/6 WT mice were purchased from SLAC Laboratory (Shanghai, China). The experimental *Park7* ^−/−^ and WT littermates were generated by crossing *Park7*‐heterozygous mice. The floxed *Park7* (*Park7* ^fl/fl^) mice (Shanghai Biomodel Organism, Shanghai, China) and the mice expressing Cre recombinase in the control of the mouse albumin promoter (Alb‐Cre; Shanghai Biomodel Organism, Shanghai, China) were applied to generate hepatocyte‐specific *Park7* knockout (*Park7* ^△hep^) mice. We used two steps to generate *Park7* ^△hep^ mice. First, a homozygous loxP‐flanked *Park7* mouse was mated with a homozygous Alb‐Cre mouse to generate the F1 mice that were heterozygous for a loxP‐flanked *Park7* allele and heterozygous for the Alb‐Cre allele. Second, we backcrossed these F1 mice to the homozygous loxP‐flanked *Park7* mice to generate the *Park7* ^△hep^, which were heterozygous for the Alb‐Cre allele and homozygous for the loxP‐flanked *Park7* allele. The homologous recombination of *Park7* ^fl/fl^ and *Pten* ^fl/fl^ mice is shown in Figure S18. The mice had a C57BL/6J background. The following primers were used for genotyping: CTCCTCTACTCCATTCTTCC and ACTCCCACCAATGAACAAAC (*Pten*), AGAACTCCACCTGCCTCTG and TGCCTCTGTAACAACCATCTG (*Park7*), CAGCATTGCTGTCACTTGGTC and ATTTGC CTGCATTACCGGTCG (Alb‐Cre).

### 4.3 Animal treatment and surgery

Male mice, 8–10 weeks of age, were subjected to PHx as previously described.^25^ Briefly, we first removed the liver from the ligaments after making a midline incision. Second, the pedicle of the left lobe was ligated (silk, 4/0) and resected. Finally, the middle lobe was ligated (silk 4/0) and resected. VO‐Ohpic (10 μg/kg, MedChemExpress, HY‐13074), the inhibitor of *Pten*, was administrated (.1 ml/10 g) to the mice by intra‐peritoneal as shown in Figure 6A for *Pten* inhibiting experiments. Dimethyl sulfoxide was used as the vehicle. Etomoxir (10 mg/kg, MedChemExpress, HY‐50202), the inhibitor of CPT1a, was administrated (.05 ml/10 g) to the mice by tail vein. PBS was used as the vehicle. AAV‐PARK7^WT^, AAV‐PARK7^C106A^ or AAV‐control were injected via the tail vein with 150 μl of PBS containing 1 × 10^12^ VG. Mice were fed normally for another 6 weeks and subjected to 2/3 PHx. At each time point after PHx, blood was collected by heart puncture in anaesthetized mice before cervical dislocation. Liver samples were then harvested immediately for an examination. In all experiments, *Park7* ^fl/fl^ littermates were used as the control. This study was performed in strict adherence to the recommendations in the Guide for the Care and Use of Laboratory Animals published by the National Institutes of Health. All animal studies were approved by the Institutional Animal Care and Use Committees of Renji Hospital and Shanghai Jiao Tong University. All authors have reviewed and approved the final manuscript and have access to the study data.

### 4.4 Statistical analysis

Data are presented as the mean ± SEM and analysed by permutation *t*‐test and Pearson correlation. Two‐sided *p* values of less than .05 were considered statistically significant. Multiple group comparisons were performed using one‐way ANOVA followed by Bonferroni's post hoc test. We also performed Welch's ANOVA for multiple group comparisons when groups had unequal variances.

## CONFLICT OF INTERESTS

The authors declare that they have no known competing financial interests or personal relationships that could have appeared to influence the work reported in this manuscript.

## Supporting information


**Figure S1** PARK7 is associated with liver regeneration following PHx.
**Figure S2** *Park7* KO delays cell cycle progression after PHx.
**Figure S3** *Park7* does not affect the priming of liver regeneration post‐PHx.
**Figure S4** *Park7* KO increases liver cholesterol/cholesterol esters levels post‐PHx.
**Figure S5** *Park7* KO increases serum NEFA levels post‐PHx.
**Figure S6** The impact of *Park7* deletion on lipid metabolism–related genes
**Figure S7** Validation of hepatic‐specific *Park7* depletion
**Figure S8** The changed serum glucose and liver glycogen in *Park7^△hep^* mice
**Figure S9** *Park7* depletion does not change the expression of genes related to lipogenesis after PHx.
**Figure S10** The expression of PPARa and CPT1a before and after PHx in detail
**Figure S11** Etomoxir treatment prevents liver/body ratios of *Park7* ^△hep^ mice from recovering to *Park7* ^fl/fl^ mice.
**Figure S12** The delayed induction of pro‐cell cycle factors in *Park7^△hep^* mice is restored in *Park7‐Pten^△hep‐DKO^* after PHx.
**Figure S13** The liver/body weight ratios before and after liver regenerative phase
**Figure S14** The mRNA levels of PPARa target genes are restored in *Park7‐Pten^△hep‐DKO^* post‐PHx.
Figure S15 The levels of β‐oxidation are inhibited in *Park7* ^△hep^ mice and restored in *Park7‐Pten* ^△hep‐DKO^ mice post‐PHx.
**Figure S16** Deficiency of *Park7* aggravates ROS generation in mice liver after 2/3 PHx.
**Figure S17** The oxidized PARK7 is responsible for PARK7‐mediated regulation of liver regeneration and hepatic β‐oxidation.
**Figure S18** Strategy to generate the compound mice

## Data Availability

The data that support the findings of this study are available from the corresponding author upon reasonable request.
