# Supplementary material for: PARK7 deficiency inhibits fatty acid β‐oxidation via PTEN to delay liver regeneration after hepatectomy
Source: Clin Transl Med. 2022 Sep 23;12(9):e1061. doi: 10.1002/ctm2.1061 (PMC9505755; doi:10.1002/ctm2.1061)
Supplement: Supplementary file 1 — Figure S1 PARK7 is associated with liver regeneration following PHx. Figure S2 Park7 KO delays cell cycle progression after PHx. Figure S3 Park7 does not affect the priming of liver regeneration post‐PHx. Figure S4 Park7 KO increases liver cholesterol/cholesterol esters levels post‐PHx. Figure S5 Park7 KO increases serum NEFA levels post‐PHx. Figure S6 The impact of Park7 deletion on lipid metabolism–related genes Figure S7 Validation of hepatic‐specific Park7 depletion Figure S8 The changed serum glucose and liver glycogen in Park7△hep mice Figure S9 Park7 depletion does not change the expression of genes related to lipogenesis after PHx. Figure S10 The expression of PPARa and CPT1a before and after PHx in detail Figure S11 Etomoxir treatment prevents liver/body ratios of Park7 △hep mice from recovering to Park7 fl/fl mice. Figure S12 The delayed induction of pro‐cell cycle factors in Park7△hep mice is restored in Park7‐Pten△hep‐DKO after PHx. Figure S13 The liver/body weight ratios before and after liver regenerative phase Figure S14 The mRNA levels of PPARa target genes are restored in Park7‐Pten△hep‐DKO post‐PHx. Figure S15 The levels of β‐oxidation are inhibited in Park7 △hep mice and restored in Park7‐Pten △hep‐DKO mice post‐PHx. Figure S16 Deficiency of Park7 aggravates ROS generation in mice liver after 2/3 PHx. Figure S17 The oxidized PARK7 is responsible for PARK7‐mediated regulation of liver regeneration and hepatic β‐oxidation. Figure S18 Strategy to generate the compound mice [file CTM2-12-e1061-s001.docx]

**Supplementary Materials**

**PARK7 deficiency inhibits fatty acid β-oxidation via PTEN to delay liver regeneration after hepatectomy**

**Short title:** Role of *Park7* in liver regeneration.

Xiaoye Qu^1,2†^, Yankai Wen^1,2†^, Junzhe Jiao^2†^, Jie Zhao^1^, Xuehua Sun^2^, Fang Wang^2^, Yueqiu Gao^2^, Weifeng Tan^1^*, Qiang Xia^1^*, Hailong Wu^3^*, Xiaoni Kong^2^*

1. Department of Liver Surgery, Renji Hospital, School of Medicine, Shanghai Jiao Tong University, Shanghai, China.
2. Central Laboratory, Department of Liver Diseases, ShuGuang Hospital Affiliated to Shanghai University of Chinese Traditional Medicine, Shanghai, China.
3. Shanghai Key Laboratory of Molecular Imaging, Shanghai University of Medicine and Health Sciences, Shanghai, China.

^†^ These authors contributed equally to this study.

**Table of contents**

Materials and methods.......................................................................................................3

Primers used in RT-PCR...................................................................................................10

Plasmid sequences...........................................................................................................11

References……………………………………………………………………………………….12

Suppl. Fig. 1......................................................................................................................13

Suppl. Fig. 2......................................................................................................................14

Suppl. Fig. 3......................................................................................................................15

Suppl. Fig. 4......................................................................................................................16

Suppl. Fig. 5......................................................................................................................17

Suppl. Fig. 6......................................................................................................................18

Suppl. Fig. 7......................................................................................................................19

Suppl. Fig. 8......................................................................................................................20

Suppl. Fig. 9......................................................................................................................21

Suppl. Fig. 10....................................................................................................................22

Suppl. Fig. 11....................................................................................................................23

Suppl. Fig. 12....................................................................................................................24

Suppl. Fig. 13....................................................................................................................25

Suppl. Fig. 14....................................................................................................................26

Suppl. Fig. 15....................................................................................................................27

Suppl. Fig. 16....................................................................................................................28

Suppl. Fig. 17....................................................................................................................29

Suppl. Fig. 18....................................................................................................................30

**Materials and Methods**

**Western Blot Analysis**

Liver tissue and cell samples were subjected to western blot analysis as we previously described[^1^](#_ENREF_1). β-actin (1:20000, Sigma-Aldrich, A3854-200UL) was used as the loading control. Rabbit anti-PCNA (1:1000, 10205-2-AP), anti-CPT1a (1:1000, 15184-1-AP), anti-LaminB1 (1:1000, 12987-1-AP), anti-FASN (1:1000, 10624-2-AP) and anti-GAPDH (1:20000, HRP-60004) were purchased from Proteintech (Wuhan, China). Rabbit anti-PPARa (1:1000, ab8934), anti-PARK7 (1:1000, ab18257), anti-oxidized PARK7 (1:1000, ab169520) were purchased from Abcam (Cambridge, US). Mouse anti-PTEN (1:1000, sc-7974) were purchased from Santa Cruz (Dallas, US). Rabbit anti-STAT3 (1:1000, 4904), anti-phospho-STAT3 (Tyr705) (1:1000, 9131), anti-ACLY (1:1000, 13390), anti-ACC1 (1:1000, 4190), anti-SCD1 (1:1000, 2794), anti-NRF2 (1:1000, 12721), anti-AKT (1:1000, 9272), anti-phospho-AKT (1:1000, 4060) were purchased from Cell Signaling Technology (Danvers, US). Cytoplasm and nucleus protein were separated using commercial assay kits (Thermo Scientific, US, 78835) according to the manufacturer’s instructions. Densitometry to determine changes in protein expression was measured using ImageJ software 1.52v (<http://imagej.nih.gov/ij>, National Institutes of Health, US). To be specific, first open the glue map to be analyzed, convert the Image into 8-bit gray map through Image Type8-bit, then remove the gray background of the glue map, select each strip and draw the peak map of the strip, and complete the calculation of the peak area. Finally, the relative expression was obtained by dividing the area of the target protein by the area of the reference protein.

**Quantitative Real-Time Polymerase Chain Reaction**

Total RNA was isolated from individual liver using commercial kits to evaluate mRNA expression. Reverse-transcribed cDNA was obtained using the Total RNA rapid extraction kit (BioTeke, China, RP4002). All primer sequences were checked in GenBank to avoid inadvertent sequence homologies. They were designed and synthesized by Sangon Biotechnology (Shanghai, China). Reactions were performed using an SYBR Green PCR master mix (Vazyme, China, Q711-02). The extracted RNA was determined using Thermo Scientific™ NanoDrop™ One microuV-VIS spectrophotometer and maintained at 260/280≥2. Relative amounts of mRNA for specific genes were calculated using the 2^−ΔΔCt^ values. β-actin expression was used as an internal control. The sequences of primer pairs are listed in supplemental materials.

**Immunohistochemistry**

Paraffin-embedded liver sections (5-μm thick) were used for immunohistochemical detection of Ki67. Tissue slides were deparaffinized and rehydrated in ethanol, and then treated with 0.5% hydrogen peroxide in methanol to block endogenous peroxidase activity. The antigen retrieval method was performed using 10 mM sodium citrate (pH 6.0). After blocking nonspecific antigens with 10% BSA in PBS for 60 min, the sections were incubated with 1:200 diluted Rabbit anti-Ki67 (Abcam, China, ab15580) at 4°C overnight. Corresponding secondary antibodies were used at room temperature for one hour, followed by diaminobenzidine (DAB) staining (Dako, China, GV825). Sections were counterstained with hematoxylin for staining of nuclei. The assessment of immunostaining was performed according to the percentage of positively-stained cells. Positive cells were counted blindly in 10 high power field (HPF)/slide (x200).

**Oil Red O staining**

For Oil Red O staining, O.C.T tissue blocks from partial hepatectomy were cut into 10-μm-thick sections. Sections were washed with distilled water, and after rinsing with 60% isopropanol and distilled water. Tissues were then stained with freshly prepared Oil Red O working solution for 6 minutes at room temperature. The working solution of Oil Red O (Sigma-Aldrich, US, O0625) was prepared by diluting a stock solution, i.e. 0.5 g of Oil Red O in 100 ml of isopropanol, with distilled water at a ratio of 3:2 (v/v). After rinsing with 60% isopropanol and washing with distilled water for 5 minutes, the tissues were stained with hematoxylin for 40 seconds. The ORO positive area was automatically recognized to work out relative area in each horizon using imageJ software 1.52v. Positive area was calculated blindly in 10 HPF/slide (x200).

**Periodic Acid-Schiff staining**

Liver PAS staining (Glycogen staining) was performed using commercial assay kits (Nanjing Jiancheng Bioengineering Institute, China, D004) according to the manufacturer’s instructions.

**Ketogenesis analyses**

To measure the ketogenic activities in vivo, mice were injected i.p. with 0.5 gkg^-1^ sodium octanoate (Sigma-Aldrich, US, C5038) at 36h after PHx. Tail-vein blood was collected at 0, 1, 2 and 4 h after the injection, and serum β-hydroxybutyrate was measured[^2^](#_ENREF_2) using the β-hydroxybutyrate colorimetric assay kit (Nanjing Jiancheng Bioengineering Institute, China, H169).

**Immunofluorescent staining**

The livers were fixed in 4% paraformaldehyde and embedded in paraffin. 5 μm liver sections were stained with hematoxylin and eosin for morphological examination. Immunofluorescence of liver sections was performed as we previously described^1^. The following primary antibodies were used: PPARa (Antibodies-online, US, ABIN285918). The secondary antibodies Alexa Fluor 488 (Thermo Scientific, US, A-11008) was used. Nuclei were counterstained with DAPI before imaging. Images were captured with the fluorescence microscope (Zeiss).

**Chromatin immunoprecipitation (ChIP).**The ChIP analysis was carried out using ChIP Assay Kit (Beyotime, China, P2078). Briefly, after treating with HGF (Sigma-Aldrich, US, SRP3300)/ EGF (Sigma-Aldrich, US, SRP3196) (50ng/ml) for 24h, AML12 cells were treated with 1% formaldehyde for 10 min to crosslink proteins and chromatin. The reaction was stopped by adding 0.125M glycine for 5 min. Cells were washed with ice-cold PBS and then resuspended with ChIP lysis buffer for 10 min. Cell lysates were centrifuged to pellet the nuclei. The cell nuclei were resuspended in nuclei lysis buffer and then subjected to sonication for 15 min. Purified chromatin was analyzed on a 1.5 % agarose gel to analyze DNA fragment size. The sheared chromatin was immunoprecipitated with NRF2 antibody (Cell Signaling Technology, US, 12721) overnight. As a control, the normal IgG was used as a replacement for the NRF2 antibody. A negative control group without DNA template was also displayed. The antibody/chromatin samples were mixed with protein A sepharose beads. Protein-DNA complexes were washed and eluted, followed by a cross-link reversal step, and the resulting DNA was purified. DNA from the immunoprecipitation reaction was examined by PCR. The primer for the NRF2-responsive region of *Park7* promoter: forward: 5’- CAGCCCCAGCACATTTTACT -3’, reverse: 5’- GGCTACATTTGCAGGTGGTT -3’.

**Reactive oxygen species assay.**2’, 7’-Dichlorofluorescin diacetate (H2DCFDA) is a cell-permeable fluorogenic probe to quantify ROS. We examined the intracellular ROS levels with the H2DCFDA probe (Sigma-Aldrich, US, D399) in the frozen sections of liver tissues from 2/3 PHx models. Briefly, liver tissues were embedded with OCT-Freeze medium. After frozen, tissues were sliced into 10-μm-thick sections and washed with ice-cold phosphate buffer (PH 7.4), subsequently incubated with the fluorescent probe H2DCFDA (25μM) at 37℃ for 30min. Fluorescence intensity was determined with an excitation wavelength of 488 nm and an emission wavelength of 526 nm. Images were captured with the fluorescence microscope (Zeiss).

**MDA detection**

Malondialdehyde (MDA) is one of the final products of polyunsaturated fatty acids peroxidation in the cells, which is commonly known as a marker of oxidative stress. For MDA detection, commercial kits were used according to the manufacturer’s instructions (Nanjing Jiancheng Bioengineering Institute, China, A003). Liver tissues were homogenized with EDTA-treated ice-cold phosphate buffer, then centrifuged at 13,000rpm for 15min at 4℃. The supernatant was used to detect the content of MDA. Total protein content was determined by BCA protein assay kit (Beyotime, China, P0012).

**ELISA and biochemical assays**

Serum IL-6 (EMC004) and TNFa (EMC102a) levels were measured with ELISA kits (NeoBioscience Technology, China) according to manufacturer’s protocol. Human PARK7 levels in serum were measured with ELISA kits (Thermo Scientific, US, EH359RB) according to manufacturer’s protocol. Hepatic ATP levels were measured using ATP assay kit (Beyotime, China, S0026B) according to manufacturer’s protocol. TG (Applygen, China, E1013), NEFA (Wako, Japan, 294-63601), cholesterol/cholesterol esters (abcam, US, ab65359), NAD^+^/NADH ratio (abcam, US, ab65348), β-hydroxybutyrate (Nanjing Jiancheng Bioengineering Institute, China, H169), malonyl CoA (antibodies-online.com, US, ABIN6973424), liver glycogen (Nanjing Jiancheng Bioengineering Institute, China, A043) and serum glucose (Nanjing Jiancheng Bioengineering Institute, China, A154) were determined using commercial assay kits according to the manufacturer’s instructions.

**Small Interfering RNA**

AML12 in 6-well plates were transfected with small interfering RNA (siRNA) against Nrf2 (GenePharma, China) using the transfection reagent Lipofectamine 3000 (Thermo Scientific, US, L3000015) according to the manufacturer’s instructions.

**Plasmids Construction and Adeno-associated virus Package**Mouse full-length *Park7* or C106 cysteine mutant PARK7 cDNA was respectively inserted into pHBAAV-TBG-MCS-P2A-zsgreen vector to obtain full-length *Park7* or mutant *Park7* expression plasmid (TBG-m-*Park7* or TBG-m-*park7*(c.G106T)). Furthermore, TBG-m-*Park7*, TBG-m-*Park7*(c.G106T) or their control vector plasmid (TBG-ZsGreen) were co-transfected with packaging plasmids pAAV-RC and pHelper into HEK293T cells to produce AAV-*Park7*, AAV-*Park7* (c.G106T) or AAV-ZsGreen virus particles, respectively. Adeno-associated virus supernatant was collected 72 hours after transfection. The recombinant *Park7* overexpression and their control adeno-associated were prepared by Hanbio (Shanghai, China).

**Primers used in RT-PCR (5’-3’)**

*Ccnd1* (F: TCAAGTGTGACCCGGACTG; R: CTCCAGAAGGGCTTCAATCTGT)

*Ccne1* (F: GGTCTGAGTTCCAAGCCCAA; R: TGGTCCGTCGAGTCTCTCTC)

*Ccna2* (F: CTCGCTGCATCAGGAAGACC; R: CCTTAAGAGGAGCAACCCGTC)

*Ccnb1* (F: AGCTGGGGCTTTCTGCTTAG; R: AGTTGGTGTCCATTCACCGT)

*Ccnb2* (F: CTATCCGGCGGGCAGTTTTA; R: TCTGAGGTTTCTTCGCCACC)

*Cdk1* (F: TACACACACGAGGTAGTGACG; R: TCTGAGTCGCCGTGGAAAAG)

*Cdk2* (F: CCTGCTTATCAATGCAGAGGG; R: TGCGGGTCACCATTTCAGC)

*Cdk4* (F: ATGGCTGCCACTCGATATGAA; R: TCCTCCATTAGGAACTCTCACAC)

*Cdk6* (F: GGCGTACCCACAGAAACCATA; R: AGGTAAGGGCCATCTGAAAACT)

*Fasn* (F: GCTGCGGAAACTTCAGGAAAT; R: AGAGACGTGTCACTCCTGGACTT)

*Acc1* (F: TGACAGACTGATCGCAGAGAAAG; R: TGGAGAGCCCCACACACA)

*Scd1* (F: TTCTTCTCTCACGTGGGTTG; R: CGGGCTTGTAGTACCTCCTC)

*Srebf1* (F: GGAGCCATGGATTGCACATT; R: GGCCCGGGAAGTCACTGT)

*Acox1* (F: TAACTTCCTCACTCGAAGCCA; R: AGTTCCATGACCCATCTCTGTC)

*Crot* (F: GAACGGACATTTCAGTACCAGG; R: CTTCATTTGCGAATGGTTTCACT)

*Cd36* (F: ATGGGCTGTGATCGGAACTG; R: GTCTTCCCAATAAGCATGTCTCC)

*Fabp4* (F: AAGGTGAAGAGCATCATAACCCT; R: TCACGCCTTTCATAACACATTCC)

*Fabp2* (F: GTGGAAAGTAGACCGGAACGA; R: CCATCCTGTGTGATTGTCAGTT)

*Fabp1* (F: ATGAACTTCTCCGGCAAGTACC; R: CTGACACCCCCTTGATGTCC)

*Fatp4* (F: ACTGTTCTCCAAGCTAGTGCT; R: GATGAAGACCCGGATGAAACG)

*Mttp* (F: AAGGCCAATATGGACATCCAGGGT; R: TGGTTATTACCACAGCCACCCGAT)

*ApoB-100*(F:AGTCTACTGGAAGCCATGAAGGG;R:AATCTGCTGAGGAAGCCTGCTCA)

*Hmgcs2* (F: GAAGAGAGCGATGCAGGAAAC; R: GTCCACATATTGGGCTGGAAA)

*Acadm* (F: AGGGTTTAGTTTTGAGTTGACGG; R: CCCCGCTTTTGTCATATTCCG)

*Slc25a20* (F: GACGAGCCGAAACCCATCAG; R: AGTCGGACCTTGACCGTGT)

*Il-6* (F: TGTTCTCTGGGAAATCGTGGA; R: TTTCTGCAAGTGCATCATCGT)

*Tnfa* (F: TTCTATGGCCCAGACCCTCA; R: TTTGCTACGACGTGGGCTAC)

*Cpt1a* (F: CTCCGCCTGAGCCATGAAG; R: CACCAGTGATGATGCCATTCT)

HGF (F: ATGTGGGGGACCAAACTTCTG; R: GGATGGCGACATGAAGCAG)

HB-EGF (F: GACTTGGAAGGGACCGATCTGGA; R: TAGGGTCAGCCCATGACACCTC)

*Park7* (F: AGCCGGGATCAAAGTCACTG; R: GGTCCCTGCGTTTTTGCATC)

β-actin (F: GGCTGTATTCCCCTCCATCG; R: CCAGTTGGTAACAATGCCATGT)

**Plasmid sequences used:**

**TBG-m-Park7** ATGGCTTCCAAAAGAGCTCTGGTCATCCTGGCCAAAGGAGCAGAGGAGA

TGGAGACAGTGATTCCTGTGGATGTCATGCGGCGAGCCGGGATCAAAGTCACTGTTGCAGGCTTGGCTGGGAAGGACCCCGTGCAGTGTAGCCGTGATGTAATGATTTGTCCAGATACCAGTCTGGAAGATGCAAAAACGCAGGGACCATACGATGTGGTGGTTCTTCCAGGAGGAAATCTGGGTGCACAGAATTTATCTGAGTCGCCTATGGTGAAGGAGATCCTCAAGGAGCAGGAGAGCAGGAAGGGCCTCATAGCTGCCATCTGTGCAGGTCCTACGGCTCTGTTGGCTCACGAAGTAGGTTTTGGATGCAAGGTCACAACACACCCACTGGCTAAGGACAAAATGATGAATGGCAGTCACTACAGCTACTCAGAGAGCCGCGTGGAGAAGGACGGCCTGATCCTCACCAGCCGCGGGCCGGGGACCAGCTTTGAGTTTGCACTAGCCATTGTGGAGGCACTCGTGGGGAAAGACATGGCCAACCAAGTGAAGGCACCGCTTGTTCTCAAAGACTAG

**TBG-m-park7(c.G106T)** ATGGCTTCCAAAAGAGCTCTGGTCATCCTGGCCAAAGGAGCAG

AGGAGATGGAGACAGTGATTCCTGTGGATGTCATGCGGCGAGCCGGGATCAAAGTCACTGTTTCAGGCTTGGCTGGGAAGGACCCCGTGCAGTGTAGCCGTGATGTAATGATTTGTCCAGATACCAGTCTGGAAGATGCAAAAACGCAGGGACCATACGATGTGGTGGTTCTTCCAGGAGGAAATCTGGGTGCACAGAATTTATCTGAGTCGCCTATGGTGAAGGAGATCCTCAAGGAGCAGGAGAGCAGGAAGGGCCTCATAGCTGCCATCTGTGCAGGTCCTACGGCTCTGTTGGCTCACGAAGTAGGTTTTGGATGCAAGGTCACAACACACCCACTGGCTAAGGACAAAATGATGAATGGCAGTCACTACAGCTACTCAGAGAGCCGCGTGGAGAAGGACGGCCTGATCCTCACCAGCCGCGGGCCGGGGACCAGCTTTGAGTTTGCACTAGCCATTGTGGAGGCACTCGTGGGGAAAGACATGGCCAACCAAGTGAAGGCACCGCTTGTTCTCAAAGACTAG

**References**

1 Wen, Y. *et al.* Defective Initiation of Liver Regeneration in Osteopontin-Deficient Mice after Partial Hepatectomy due to Insufficient Activation of IL-6/Stat3 Pathway. *International journal of biological sciences* **11**, 1236-1247, doi:10.7150/ijbs.12118 (2015).

2 Wolfrum, C., Asilmaz, E., Luca, E., Friedman, J. & Stoffel, M. Foxa2 regulates lipid metabolism and ketogenesis in the liver during fasting and in diabetes. *Nature* **432**, 1027-1032, doi:10.1038/nature03047 (2004).

**Supplemental Figures**

Figure S1

**
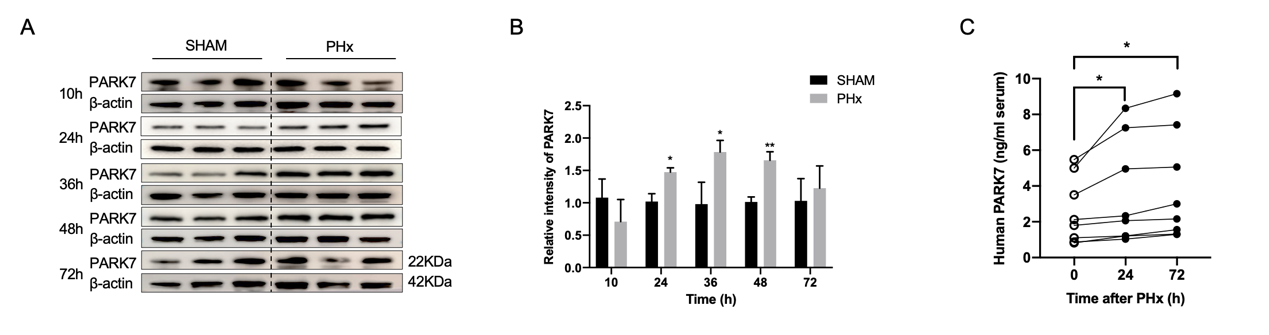
**

**Supplemental figure 1. PARK7 is associated with liver regeneration following PHx.** (A) Immunoblot of PARK7 at indicated time points after PHx was performed (n = 4–6 mice/group). β-actin was used as a loading control. (B) PARK7 expression was quantified. Representative of three experiments. (C) Human serum PARK7 levels in normal donors at the timepoints of 24 and 72 hours after left lateral lobectomy were examined (n=7 patients). Data are shown as mean ± SEM. *P<0.05; **P<0.01.

Figure S2


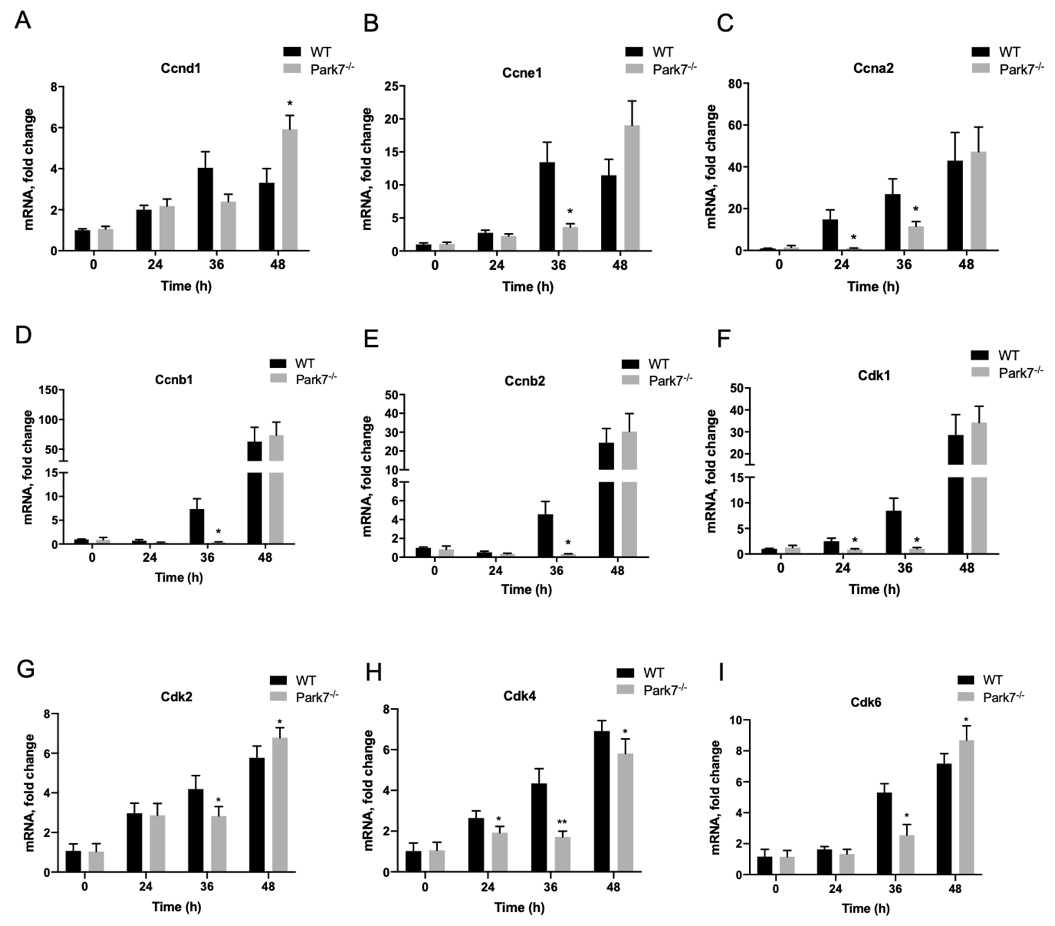


**Supplemental figure 2. *Park7* KO** **delays cell cycle progression after PHx.** (A-I) mRNA levels of cell-cycle associated molecules including *Ccnd1*, *Ccne1,* *Ccna2*, *Ccnb1*, *Ccnb2*, *Cdk1*, *Cdk2*, *Cdk4* and *Cdk6* at indicated time points after PHx were determined (n=3-4 mice/group). Data are shown as mean ± SEM. *P<0.05; **P<0.01.

Figure S3

**
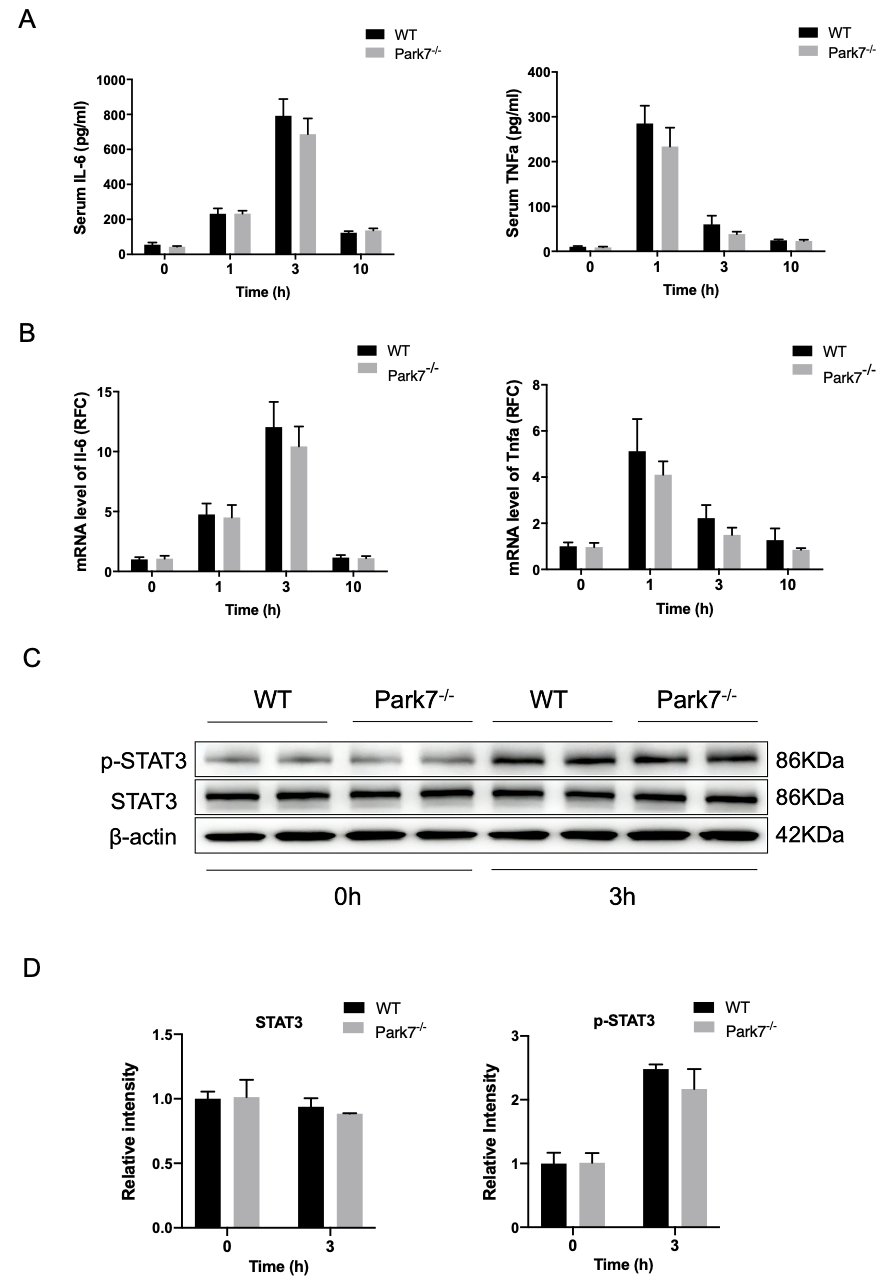
**

**Supplemental figure 3. *Park7* does not affect the priming of liver regeneration post PHx.** WT and *Park7^-/-^* mice were subjected to PHx. (A) Serum IL-6 and TNFa levels were determined at indicated time points after PHx (n=4-6 mice/group). (B) mRNA expressions of *Il6* and *Tnfa* were determined at indicated time points after PHx (n=3-4 mice/group). (C) Immunoblot of phosphorylated STAT3 and STAT3 at indicated time points after PHx was performed (n = 4–6 mice/group). β-actin was used as a loading control. (D) Phosphorylated STAT3 and STAT3 levels were quantified. Representative of three experiments. Data are shown as mean ± SEM.

Figure S4

**
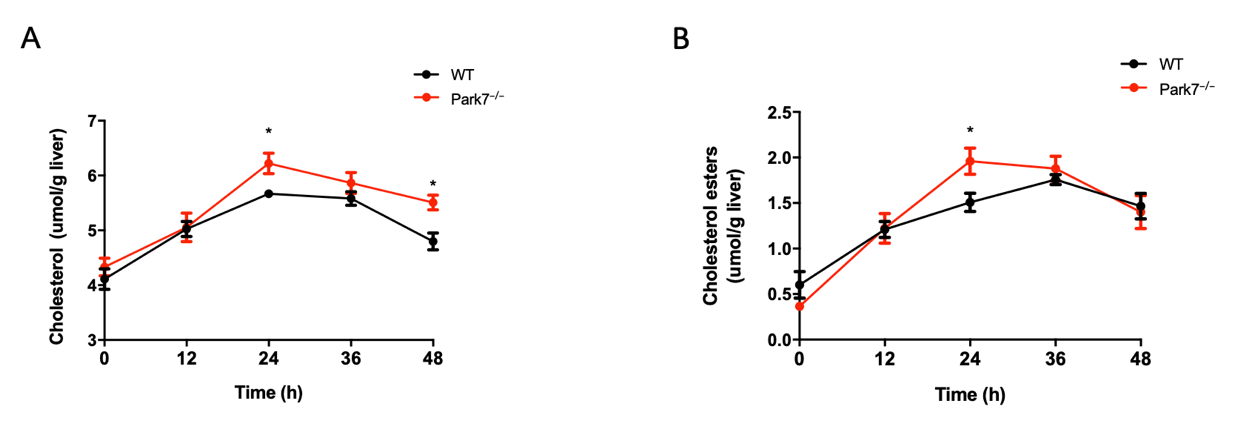
**

**Supplemental figure 4. *Park7* KO increases liver Cholesterol/Cholesterol esters levels post PHx.** WT and *Park7*^-/^*^-^* mice were subjected to PHx. (A) Liver cholesterol levels were determined at indicated time points after PHx (n=4-6 mice/group). (B) Liver cholesterol esters levels were determined at indicated time points after PHx (n=4-6 mice/group). Data are shown as mean ± SEM. *P<0.05.

Figure S5


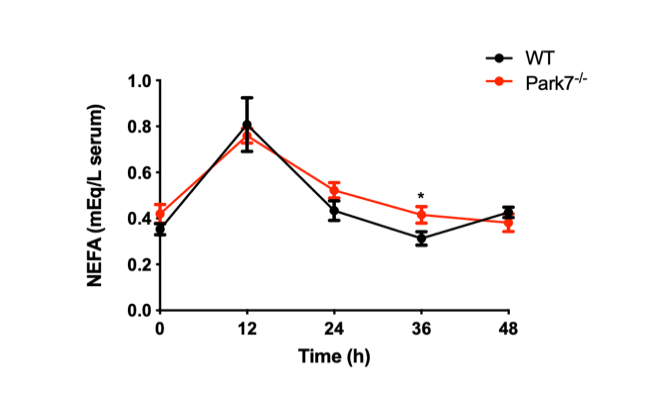


**Supplemental figure 5.** ***Park7* KO increases serum NEFA levels post PHx.** Serum NEFA levels were determined at indicated time points after PHx (n=4-6 mice/group). Data are shown as mean± SEM. *P<0.05.

Figure S6


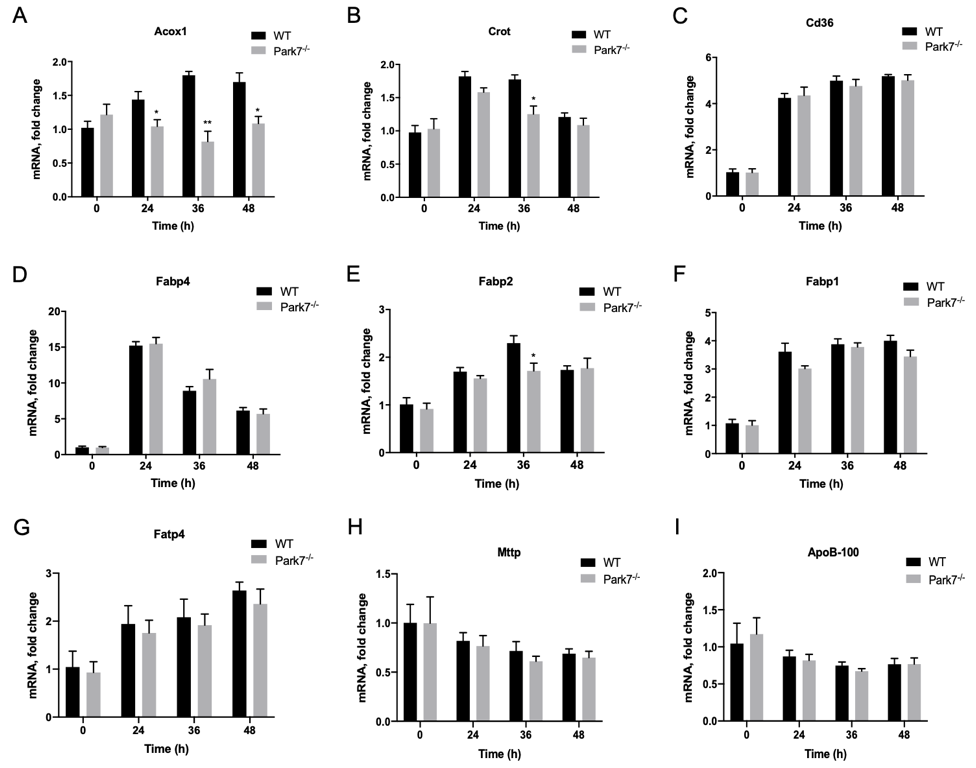


**Supplemental figure 6. The impact of *Park7* deletion on lipid metabolism related genes.** WT and *Park7*^-/-^ mice were subjected to PHx. (A-B) mRNA levels of peroxisome β-oxidation related genes at the indicated times after PHx were determined by qPCR assays (n=3-4 mice/group). (C-G) mRNA levels of lipid import related genes at the indicated times after PHx were determined (n=3-4 mice/group). (H-I) mRNA levels of VLDL assembly and excretion related genes at the indicated times after PHx were determined (n=3-4 mice/group). Data are shown as mean± SEM. *P<0.05; **P<0.01.

Figure S7


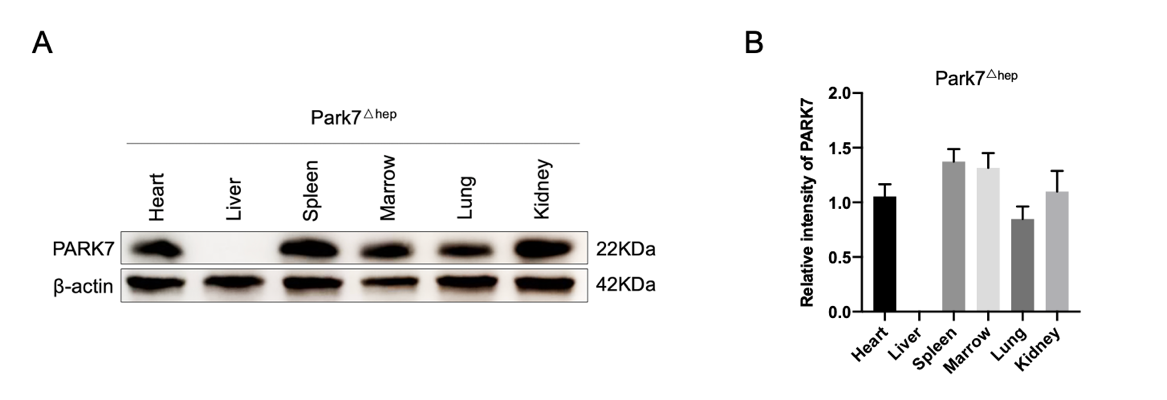


**Supplemental figure 7.** **Validation of hepatic specific *Park7* depletion.** (A) Immunoblot of PARK7 in indicated organs of *Park7^△hep^* mice to confirm hepatic specific depletion of *Park7* (n = 4–6 mice/group). β-actin was used as a loading control. (B) PARK7 expression was quantified. Representative of three experiments.

Figure S8


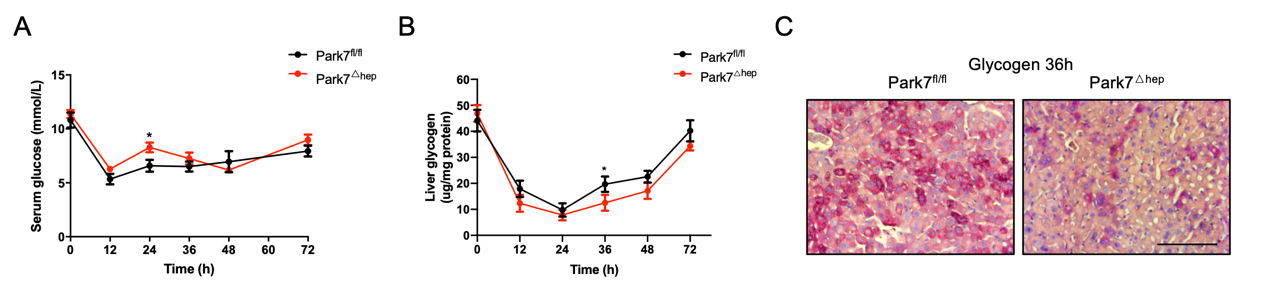


**Supplemental figure 8.** **The changed serum glucose and liver glycogen in *Park7^△hep^* mice.** *Park7*^fl/fl^ and *Park7^△hep^* mice were subjected to PHx. (A) Serum glucose was determined at the indicated times after PHx (n=4-6 mice/group). (B) Liver glycogen was determined at the indicated times after PHx (n=4-6 mice/group). (C) PAS staining was performed in *Park7*^fl/fl^ and *Park7^△hep^* mice at 36h after PHx. Data are shown as mean ± SEM. *P<0.05. Scale bar: 100µm.

Figure S9


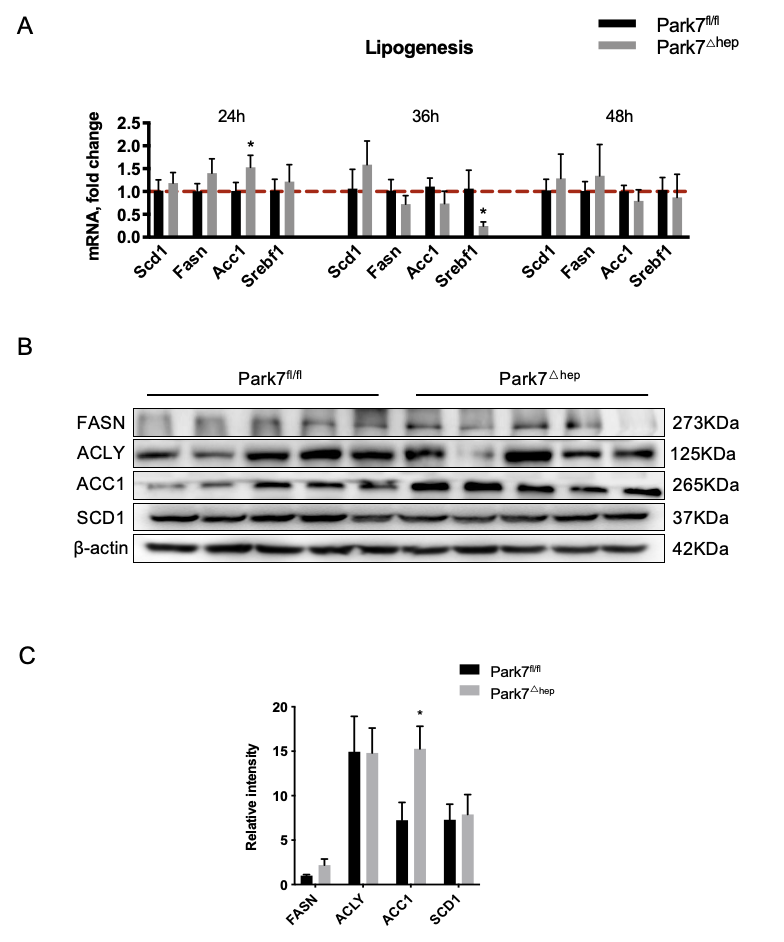


**Supplemental figure 9. *Park7* depletion does not change the expression of genes related to lipogenesis after PHx.** *Park7^fl/fl^* and *Park7^△hep^* mice were subjected to PHx. (A) mRNA levels of *Scd1*, *Fasn*, *Acc1*, and *Srebf1* at the indicated times after PHx were determined by qPCR assays (n=3-4 mice/group). (B) Immunoblot of FASN, ACLY, ACC1, SCD1 at 36 hours after PHx was performed (n = 4–6 mice/group). β-actin was used as a loading control. (C) Related protein expression was quantified. Representative of three experiments. Data are shown as mean ± SEM. *P<0.05.

Figure S10

**
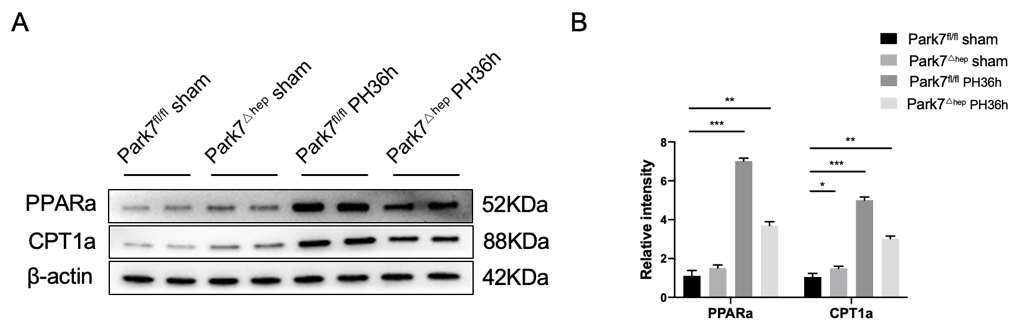
**

**Supplemental figure 10. The expression of PPARa and CPT1a before and after PHx in detail.** (A) Immunoblot of PPARa and CPT1a in *Park7*^fl/fl^ sham, *Park7*^△hep^ sham，*Park7*^fl/fl^ PH36h and *Park7*^△hep^ PH36h mice was performed (n = 4–6 mice/group). β-actin was used as a loading control. (B) PPARa and CPT1a expression was quantified. Representative of three experiments. Data are shown as mean ± SEM. *P<0.05; **P<0.01; ***P<0.001.

Figure S11


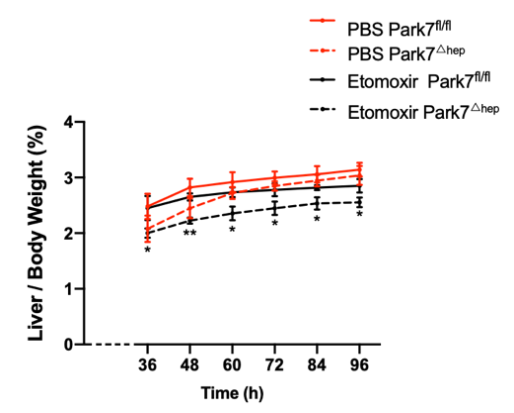


**Supplemental figure 11. Etomoxir treatment prevents liver/body ratios of** ***Park7*^△hep^ mice from recovering to *Park7*^fl/fl^ mice.** Liver/body weight ratios were calculated at indicated time points in PBS *Park7^fl/fl^*, PBS *Park7^△hep^*, etomoxir *Park7^fl/fl^* and etomoxir *Park7^△hep^* mice, respectively (n=4-6 mice/group). Data are shown as mean ± SEM. *P<0.05; **P<0.01.

Figure S12


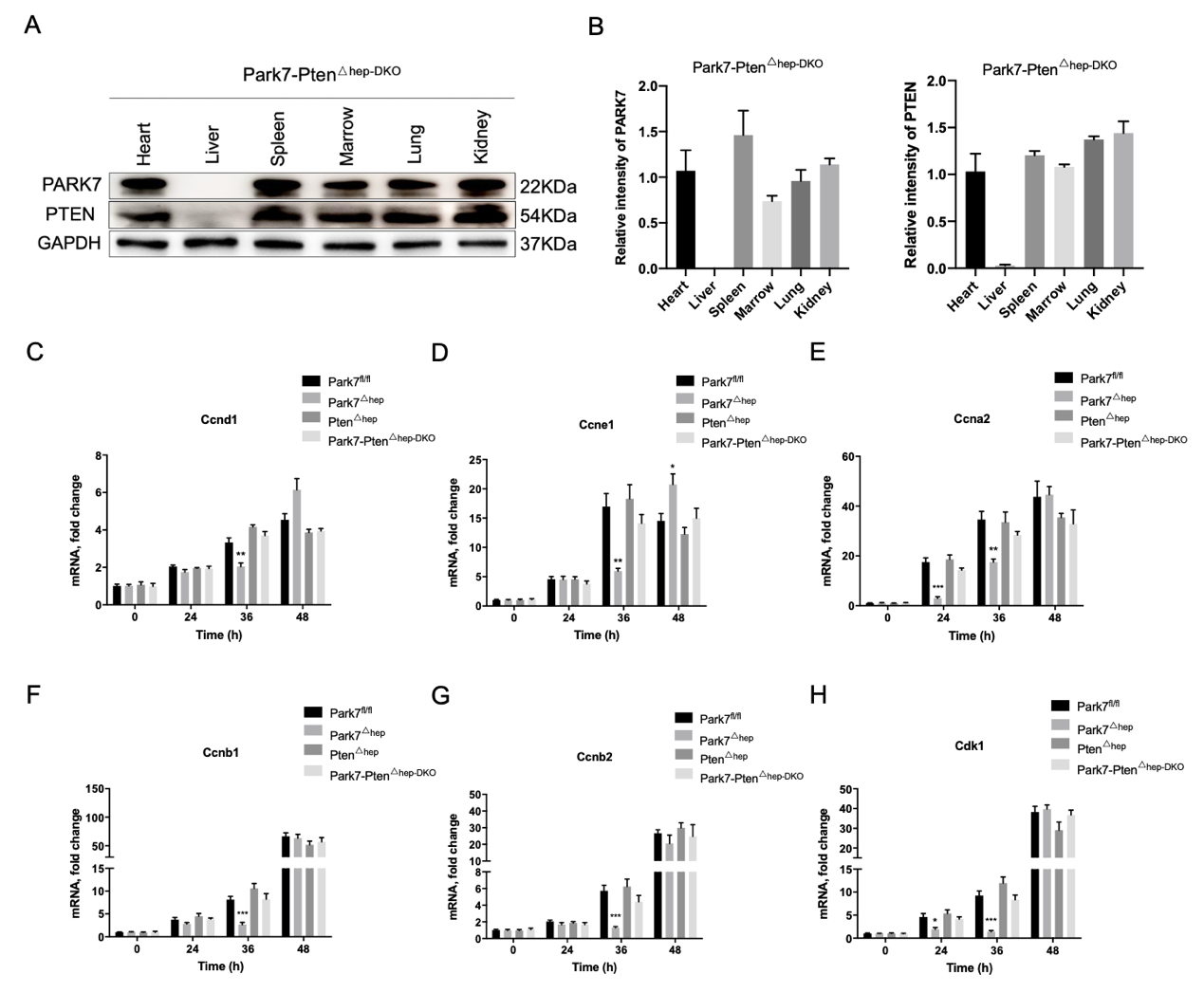


**Supplemental figure 12. The delayed induction of pro-cell cycle factors in *Park7^△hep^* mice is restored in *Park7-Pten^△hep-DKO^* after PHx.** (A) PARK7 and PTEN immunoblot in each organ of *Park7-Pten*^△hep-DKO^ mice was performed (n = 4–6 mice/group). β-actin was used as a loading control. (B) PARK7 and PTEN expression was quantified. Representative of three experiments. (C-E) mRNA expression of *Ccnd1*, *Ccne1* and *Ccna2* at indicated time points after PHx were determined (n=3-4 mice/group). (F-H) mRNA expression of *Ccnb1*, *Ccnb2* and *Cdk1* at indicated time points after PHx were determined (n=3-4 mice/group). Data are shown as mean ± SEM. *P<0.05; **P<0.01; ***P<0.001.

Figure S13


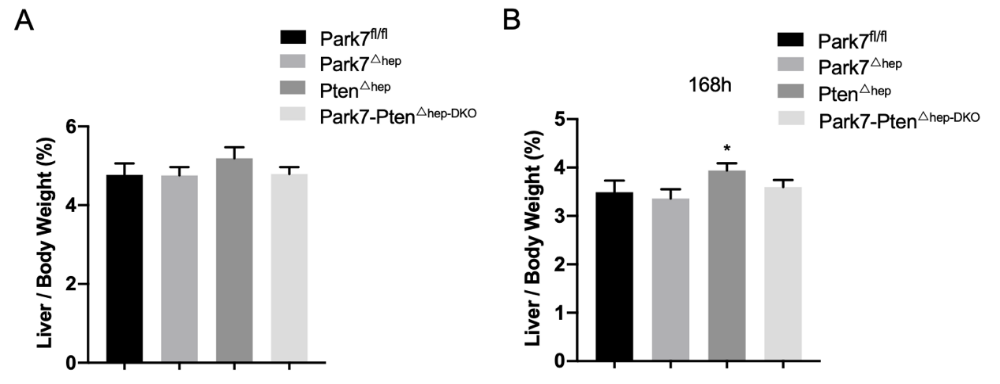


**Supplemental figure 13.** **The liver/body weight ratios before and after liver regenerative phase.** (A) Liver/body weight ratios were calculated prior to PHx in *Park7*^fl/fl^, *Park7*^△hep^, *Pten*^△hep^ and *Park7-Pten*^△hep-DKO^ mice (n=4-6 mice/group). (B) Liver/body weight ratios were calculated at 168h after PHx in *Park7*^fl/fl^, *Park7*^△hep^, *Pten*^△hep^ and *Park7-Pten*^△hep-DKO^ mice (n=4-6 mice/group). Data are shown as mean ± SEM. *P<0.05.

Figure S14


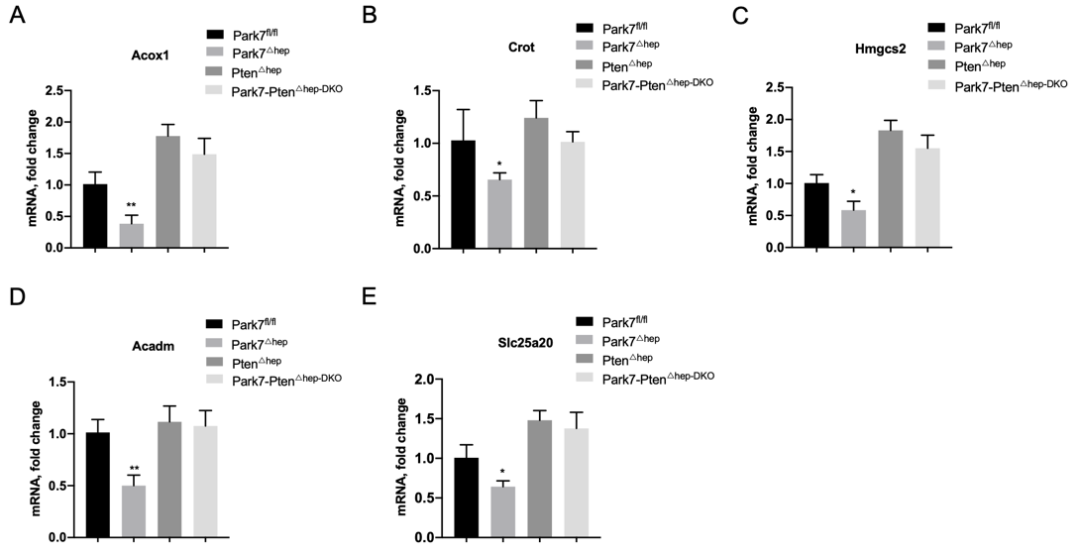


**Supplemental figure 14. The mRNA levels of PPARa target genes are restored in *Park7-Pten^△hep-DKO^* post PHx.** (A-C) The mRNA levels of PPARa target genes such as *Acox1*, *Crot* and *Hmgcs2* at 36h after PHx were determined (n=3-4 mice/group). (D-E) The mRNA levels of other β-oxidation associated genes such as *Acadm* and *Slc25a20* at 36h after PHx were determined (n=3-4 mice/group). Data are shown as mean ± SEM. *P<0.05; **P<0.01.

Figure S15


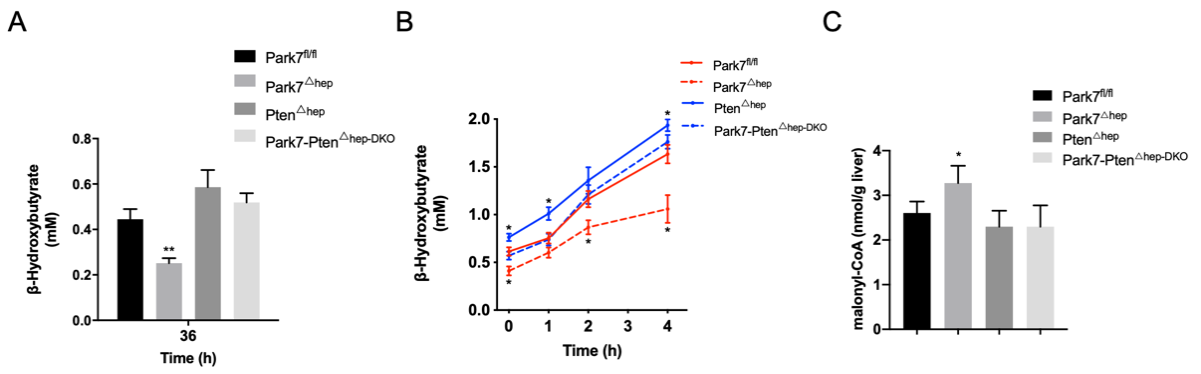


**Supplemental figure 15. The levels of β-oxidation are inhibited in *Park7*^△hep^ mice and restored in *Park7-Pten*^△hep-DKO^ mice post PHx.** (A) Serum β-hydroxybutyrate was measured at 36h post-PHx (n=3-4 mice/group). (B) 36h post-PHx mice were injected i.p. with 0.5 gkg-1 sodium octanoate. Serum β-hydroxybutyrate was measured from tail-vein blood at the indicated time points (n=3-4 mice/group). (C) Hepatic malonyl CoA levels were detected at 36h post PHx in *Park7*^fl/fl^, *Park7*^△hep^, *Pten*^△hep^ and *Park7-Pten*^△hep-DKO^ mice (n=3-4 mice/group). Data are shown as mean ± SEM. *P<0.05; **P<0.01.

Figure S16


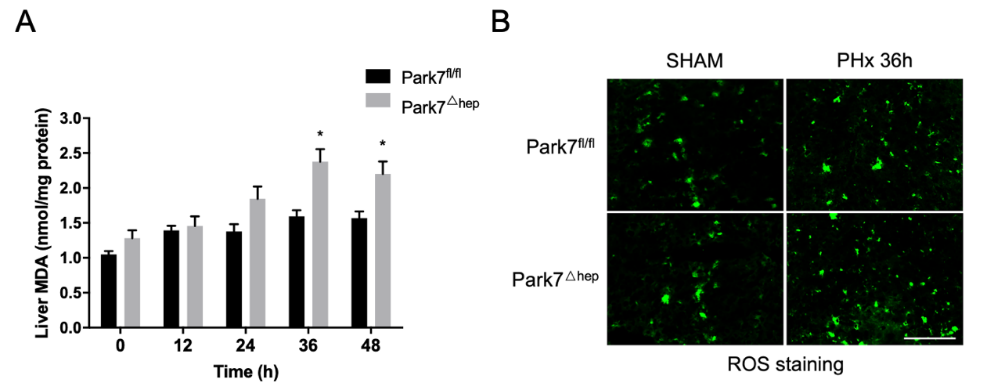


**Supplemental figure 16. Deficiency of *Park7* aggravates ROS generation in mice liver after 2/3 PHx.** (A) MDA content in liver tissue homogenates was detected in *Park7*^fl/fl^ and *Park7*^△hep^ mice at indicated time points (n=3-4 mice/group). (B) ROS production was visualized by H2DCFDA probe in frozen liver sections from *Park7*^fl/fl^ mice and *Park7*^△hep^ mice. Scale bar: 100µm. Data are shown as mean ± SEM. *P<0.05.

Figure S17


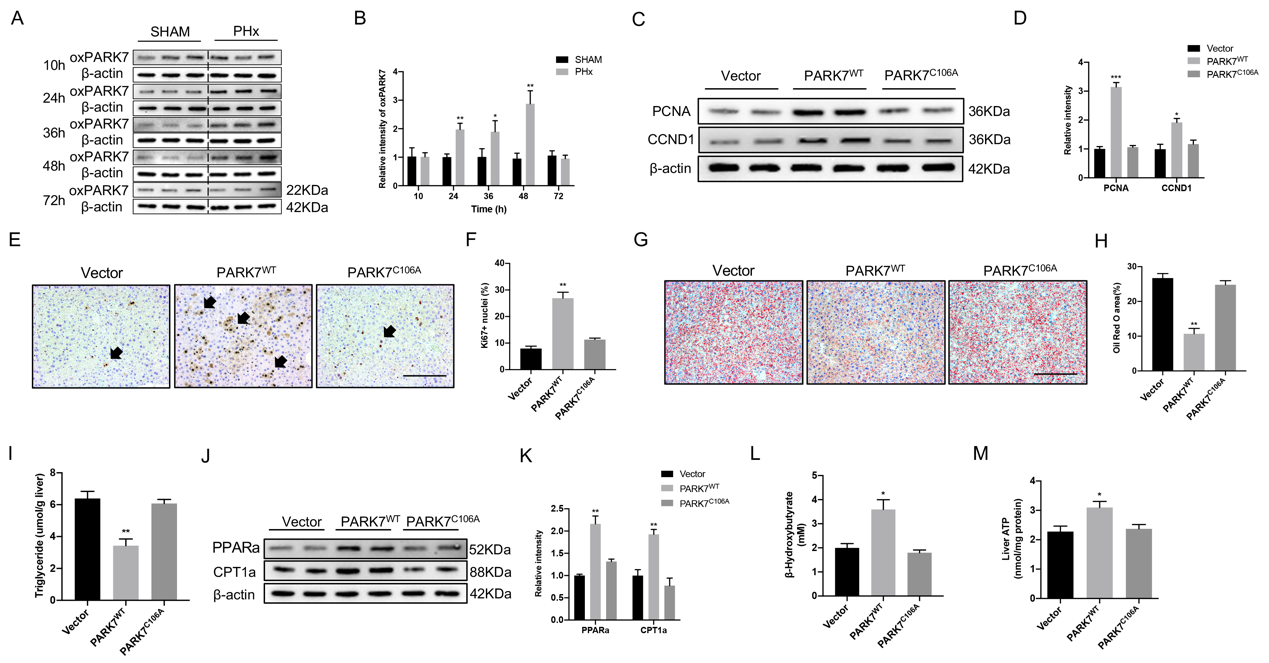


**Supplemental figure 17. The oxidized PARK7 is responsible for PARK7 mediated regulation of liver regeneration and hepatic β-oxidation.** The AAV of control vector, PARK7^WT^ and PARK7^C106A^ (1X10^12^ VG) were administrated to *Park7*^△hep^ mice (4-6 mice/group) by tail vein. (A) Immunoblot of oxPARK7 at indicated time points after PHx was performed (n = 4–6 mice/group). β-actin was used as a loading control. (B) oxPARK7 expression was quantified. Representative of three experiments. (C) Immunoblot of PCNA/CCND1 at 36h after PHx was performed (n = 4–6 mice/group). β-actin was used as a loading control. (D) PCNA/CCND1 expression was quantified. Representative of three experiments. (E) Immunohistochemistry of hepatic Ki67 at 36 hours after PHx was performed. (F) Percentages of Ki67 positive hepatocytes were calculated (n=4-6 mice/group). (G) Oil Red O staining of hepatic lipid accumulation was performed at 36h post PHx. (H) The areas of positive staining were quantified (n=4-6 mice/group). (I) Hepatic TG was measured at 36h after PHx (n=4-6 mice/group). (J) Immunoblot of hepatic PPARa and CPT1a at 36h after PHx was performed in Vector, PARK7^WT^ and PARK7^C160A^ mice (n = 4–6 mice/group). β-actin was used as a loading control. (K) PPARa and CPT1a expression was quantified. Representative of three experiments. (L) Serum β-hydroxybutyrate was measured at 36h after PHx (n=3-4 mice/group). (M) Biochemical detection of hepatic ATP levels at 36h post 2/3 PHx (n=3-4 mice/group). Data are shown as mean± SEM. *P<0.05; **P<0.01; ***P<0.001. Scale bar: 100µm.Figure S18


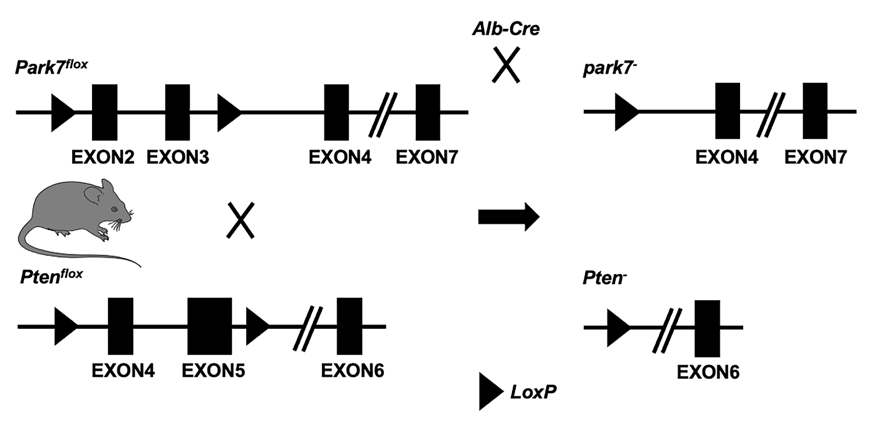


**Supplemental figure 18. Strategy to generate the compound mice.** As showed, *Park7*^fl/fl^ mice with exons 2 and 3 of Park7 flanked by loxP sites by homologous recombination were mated with mice carrying the Cre transgene under the control of the albumin promoter (Alb-Cre). *Pten*^fl/fl^ mice with exons 4 and 5 of Pten flanked by loxP sites by homologous recombination were mated with mice carrying the Cre transgene under the control of the albumin promoter.
